# Determining Plant – Leaf Miner – Parasitoid Interactions: A DNA Barcoding Approach

**DOI:** 10.1371/journal.pone.0117872

**Published:** 2015-02-24

**Authors:** Stéphane A. P. Derocles, Darren M. Evans, Paul C. Nichols, S. Aifionn Evans, David H. Lunt

**Affiliations:** School of Biological, Biomedical and Environmental Sciences, University of Hull, Hull, United Kingdom; Institut National de la Recherche Agronomique (INRA), FRANCE

## Abstract

A major challenge in network ecology is to describe the full-range of species interactions in a community to create highly-resolved food-webs. We developed a molecular approach based on DNA full barcoding and mini-barcoding to describe difficult to observe plant – leaf miner – parasitoid interactions, consisting of animals commonly regarded as agricultural pests and their natural enemies. We tested the ability of universal primers to amplify the remaining DNA inside leaf miner mines after the emergence of the insect. We compared the results of a) morphological identification of adult specimens; b) identification based on the shape of the mines; c) the COI Mini-barcode (130 bp) and d) the COI full barcode (658 bp) fragments to accurately identify the leaf-miner species. We used the molecular approach to build and analyse a tri-partite ecological network of plant – leaf miner – parasitoid interactions. We were able to detect the DNA of leaf-mining insects within their feeding mines on a range of host plants using mini-barcoding primers: 6% for the leaves collected empty and 33% success after we observed the emergence of the leaf miner. We suggest that the low amplification success of leaf mines collected empty was mainly due to the time since the adult emerged and discuss methodological improvements. Nevertheless our approach provided new species-interaction data for the ecological network. We found that the 130 bp fragment is variable enough to identify all the species included in this study. Both COI fragments reveal that some leaf miner species could be composed of cryptic species. The network built using the molecular approach was more accurate in describing tri-partite interactions compared with traditional approaches based on morphological criteria.

## Introduction

The past decade has seen significant advances in our understanding of complex species interaction networks (see [1, 2] for reviews). Ecological networks describe the interactions between species, the underlying structure of communities and the function and stability of ecosystems [3, 4, 5, 6, 7, 8]. Recently, there has been considerable interest in the development and application of ecological network analysis for understanding species invasions and biological control, particularly in agro-ecosystems [9, 10]. For example in 2008, Bukovinsky et al. [5] used an ecological network approach to demonstrate the direct and indirect impacts of plant quality on the structure of host – parasitoid networks. In 2009, Macfadyen et al. [11] used the same approach to study natural pest-control in farmland ecosystems and showed significant structural differences in host – parasitoid networks between conventional and organic farms.

A major challenge in all of these studies is to fully describe the complete range of species interactions in a community in order to create highly-resolved ecological networks [12]. To date, most host – parasitoid networks have been constructed using traditional insect rearing practices in controlled laboratory conditions (e.g. [7]). Molecular diagnostic approaches such as DNA barcoding and other emerging DNA-based methods [13] have become a powerful tool to quantify species interactions in a range of invertebrate communities (e.g. [14, 15, 16], for a review see [13]). These molecular approaches have recently been used to describe host – parasitoid ecological networks with unprecedented precision. In 2008 for example, Traugott et al. [17] used multiplex PCRs to exhaustively study the entire parasitoid community of the pest aphid *Sitobion avenae* (F.), and their approach described the interactions between both primary and secondary parsitoids that had previously been very difficult to elucidate using traditional rearing methods. Derocles et al. [16] developed this approach further and used a novel parasitoid group-specific primers pair to create an ecological network consisting of more than 60 aphid and parasitoid species and showed high network compartmentalisation between farmland crop and non-crop environments. Recent work by Wirta et al. [18] demonstrated the value of using molecular tools to construct and analyse highly resolved host – parasitoid networks, without which an important number of species and interactions would have been missed using traditional rearing methods.

Leaf miners are phytophagous insects, mainly consisting of flies (Diptera) moths (Lepidoptera) and beetles (Coleoptera). Many leaf mining insects are considered to be agricultural pests and are therefore economically important organisms [19, 20, 21]. Leaf miners attack a very wide range of terrestrial plants, including cultivated plants, by feeding within leaves: adult females oviposite one (or several) egg either on the leaf surface or by puncturing the leaf. Hatched larvae feed on leaf tissue making a tunnel or a blotch (hereafter termed a “mine”). However, despite their potential impact on a range of commercially important crops, they are poorly studied organisms, partly due to their cryptic life-cycle [22] which can create major challenges when attempting to incorporate leaf mining insects into complex ecological networks. Pocock et al. [7] and Evans et al. [8] for example constructed a highly resolved ‘network of ecological networks’ which was unable to fully integrate plant – leaf miner interactions due to the difficulties of identifying species based on laboratory rearing and mine morphology. Indeed, leaf miners are difficult to rear in the laboratory, mainly because collecting infested leaves and rearing adults from larvae within the mines often results in premature death. Moreover, a considerable number of sampled leaves, which appear infested, are often empty on closer inspection because the leaf miner has already emerged as an adult. Another problem occurs when it is not the leaf mining insect adult which emerges from the infested leaf but a Hymenoptera parasitoid of the leaf miner, rendering the identification of the leaf miner host virtually impossible. Finally, there are considerable taxonomic challenges in identifying leaf mining insects as they belong to a diverse range of dipteran and lepidopteran families. Even with taxonomic expertise, the existence of cryptic species within several leaf miner families leads to misidentifications and to a potentially biased description of the plant – leaf miner – parasitoid interaction networks [14, 23]. A solution to some of these problems would be to use the shape of the mine made by the insect when it feeds within the leaf as criteria to identify the leaf miner [24], however, the reliability of this approach still needs to be demonstrated and this method often fails to identify to the species-level.

In this paper, our objective is to overcome these problems through the development of a novel molecular approach that can be used to construct precise plant – leaf miner interactions based on remant DNA within the mines. We hypothesise that the DNA of leaf mining insects from leaf mines can be used to identify the phytophagous insects through DNA barcoding. We predict that such DNA is potentially available if a) the fly or moth larva is present in the mine; b) whether or not the leaf mining insect was parasitized, and c) even if the leaf miner has emerged as an adult, by leaving behind remnant or DNA. Since the remaining DNA of the leaf miner comes from cells shed during feeding, waste excretion, and the development of the insect, and is of unknown age, it is likely that this DNA is degraded. In this context a classic DNA full barcoding approach is potentially inefficient because the 658 bp fragment is too long to be amplified [25]. Consequently, there is a need to use smaller markers to be able to amplify the degraded DNA and to then identify the leaf mining insects [26, 27, 28]. This approach, while potentially suitable for some degraded DNA, is unlikely to work ubiquitously as the older and more highly degraded samples are likely to be unavailable for any standard PCR amplification. Here, we develop a “minibarcoding” approach that employs a fragment of 130 bp to reduce the problem of degraded DNA [27, 28]. This fragment seems to be variable enough to identify a large number of vertebrate and invertebrate specimens to the species-level [28]. Indeed, this approach has been used to determine the prey of insectivorous bats using the remaining DNA in their faeces [29]. We selected as well the primers developed in these recent studies [28, 29] to amplify the degraded DNA within the mines. Indeed these primers has been designed [28, 29] and successfully tested on a broad diversity of arthropods [29], including Diptera and Lepidoptera.

In this study, we develop for the first time a molecular approach using minibarcoding to amplify and sequence the remaining Dipteran and Lepidopteran DNA within the mines made by leaf mining insects in an agricultural environment. We compare the ability of 130 bp minibarcoding to accurately identify the leaf miner specimens with 1) the shape of the mine as criteria of identification and 2) the classic 658 bp full barcode fragment. We then show how our molecular approach, while failing to amplify from what we speculate are the older environmental samples, can still be used to recover valuable ecological data to construct a tri-partite ecological network consisting of plants, leaf mining insects and their parasitoids, and compare the structure with a network based on traditional insect rearing.

## Materials and Methods

### Ethics statement

This study was approved and carried out in strict accordance with the University of Hull’s Ethical Review Committee (Permit Number: U036). Focal species (leaf-mining caterpillars) are regarded as agricultural pests within the UK and controlled though pesticide spraying as part of routine farm management. Non-target parasitoid wasps can be affected in a similar manner. All plant and insect samples were collected from Stockbridge Technology Centre, an independent horticultural centre of excellence supported by both the production and supply sectors of the industry. None of the species are protected under UK/EU law thus no permits were required.

### Taxonomic sampling

Between 13^th^ June and 15^th^ October 2012, 144 transects of 9 meters by 2 meters were sampled in a range of agricultural habitats at Stockbridge Technology Centre, Cawood, United Kingdom, (Lat 53.824854, Lon -1.150554) as part of an on-going research project. We systematically collected 407 infested leaves from 16 host plant species. Each infested leaf was stored separately in a hermetic sealed plastic bag at 20°C. We carried out microscopic inspection to determine whether or not an insect was present. If the mine was empty, we cut out the mine with a sterile scalpel and stored it at -20°C. For insects present, we waited up to fourteen days for insect emergence from the infested leaves, and if no insect emerged, we dissected the leaf to extract the remaining larva. A photograph of each infested leaf was made in order to attempt to identify the leaf mining insect based on the shape of the mine. After a dissection of an immature stage or an emergence, we cut out the mine with a sterile scalpel and stored it at -20°C. Emerging adults and larvae were preserved in 96% ethanol and stored at -20°C in the laboratory.

We attempted to identify the leaf mining insects based on the shape of the mine in the leaf using online reference libraries: www.leafmines.co.uk and www.ukflymines.co.uk. Emerged dipteran and hymenopteran adults were identified by taxonomists at the National Museum of Wales and the Natural History Museum London. Diptera identification included the dissection of male genitalia and the use of plant identity. Because no adult Lepidoptera emerged, specimens were only at immature stages and the morphological identification of the insects was not possible. The specimens used in the study, including information pertaining to host plant and sampling date are summarized in S1 Table.

### DNA extraction, amplification and sequencing

For adult insects and immature specimens dissected, a non-invasive protocol of DNA extraction was used, allowing a morphological re-examination following sequencing [30]. DNA was extracted from single individual using the Qiagen DNeasy kit following the manufacturer’s protocol. To extract the DNA left behind within the mines by leaf mining insects, we used a salting out protocol [31] on the mines. The mine, cut with a sterile scalpel, was put in a 1.5 mL microfuge tube and incubated at 37°C overnight in 600 μL TNES with 100 μg/mL Proteinase K. Proteins were precipitated with 150 μL 5M NaCl and hard shaking performed manually for 15 sec and pelleted in a microfuge at 12,000 rpm for 5 min. DNA was then precipitated from the decanted supernatant with 1 volume 100% ethanol, pelleted, washed in 70% ethanol, air-dried, and dissolved in 50 μL sterile water. DNA in sterile water was used as the template DNA in polymerase chain reactions (PCR).

The DNA full Barcode fragment of cytochrome c oxidase subunit I (COI) was amplified from each insect specimen (adults and immatures) and mine extractions using the primers: LCO1490: 5'-GGTCAACAAATCATAAAGATATTGG-3' and HCO2198: 5'-TAAACTTCAGGGTGACCAAAAAATCA-3' [32]. The thermocycling protocol was: 94°C for 180 sec; 37 cycles of 94°C for 30 sec, 50°C for 60 sec, 72°C for 90 sec; with a final cycle of 72°C for 600 sec.

The DNA Mini-barcode fragment of COI was amplified using the following primers [28] on adults and immatures Diptera and Lepidoptera and on each mine: Uni-MinibarR1: 5'-GAAAATCATAATGAAGGCATGAGC-3' and Uni-MinibarF1: 5'-TCCACTAATCACAARGATATTGGTAC-3' using the thermocycling protocol specified by Meusnier et al. [28].

A second DNA Mini-barcode fragment overlapping the same COI region was amplified using the following primers [29] on adults and immatures Diptera and Lepidoptera and on each mine: ZBJ-ArtF1c: 5’-AGATATTGGAACWTTATATTTTATTTTTGG-3’ ZBJ-ArtR2c: 5’-WACTAATCAATTWCCAAATCCTCC-3’ using the thermocycling protocol specified by Zeale et al. [29].

The 25 μl PCR mixtures contained 1 X Qiagen enzyme buffer (containing 1.5 mM MgCl_2_), 1 unit of Taq polymerase, 17.5 pmol of each primer, 25 nM of each dNTP and 2 μl of DNA extract.

After a positive PCR amplification, PCR products were purified and sequenced on both strands. Bioedit 7.2.5 [33] was used for read assembly and sequences were aligned using Clustal W 1.81 with default settings [34]. Alignments were translated to amino acids using MEGA version 5 [35] to detect frame shift mutations and premature stop codons, which may indicate the presence of nuclear copies of mitochondrial DNA.

### Data analysis

We compared three methods of DNA-based taxonomic identification. First, we used megaBLAST [36] to identify the best BLAST hit based on the two fragments of COI amplified (COI full barcode with LCO1490-HCO2198 primers; COI minibarcode with either Uni-MinibarF1-Uni-MinibarR1 or ZBJ-ArtF1c-ZBJ-ArtR1c). Next, we used the sequence identification approach implemented in BOLD [37]. Finally, we conducted maximum likelihood (ML) phylogenetic analyses using RAxML 7.0.4 [38] using the target sequences and the top 20 hits found with megaBLAST and any additional taxa from the top 20 hits found with BOLD animal identification.

The branch lengths of the Maximum Likelihood tree represent the divergence of the fragments sequenced. We calculated pairwise intraspecific sequence divergences with MEGA version 5 (Substitution type: nucleotide; Model: K2P; species were identified with megaBLAST, animal identification in BOLD and ML analysis).

### Ecological network

Plant – leaf miner – parasitoid interactions were constructed using: 1) Morphological identification of the adult leaf miners and adult parasitoids. If adult specimens were not available, we relied on the identification based on the shape of the mine; 2) Molecular identification based on COI sequences from the emerged adult parasitoids, from the emerged leaf miners and/or from the remaining DNA within the leaf mines.

We then compared the plant – leaf miner – parasitoid food web constructed with the molecular identifications of species with a plant – leaf miner – parasitoid food web constructed only with morphological identifications.

For network analysis, we used the following commonly used network metrics in our comparisons: The **number of links** is the total number of plant – leaf miner – parasitoid interactions found in the food web. The **qualitative link density** is the total number of trophic links divided by the total number of species; the **quantitative link density** is the average of numbers of prey and of consumers [39]. The **qualitative Vulnerability** is calculated as the total number of trophic links divided by the number of leaf miner and plant species; the **quantative vulnerability** is the average effective consumer taxa overall all prey taxa [39]. The qualitative **Generality** is calculated as the total number of trophic links divided by the number of parasitoid and leaf miner species; the **quantitative Generality** is the average effective prey taxa overall all consumer taxa [39]. We defined **connectance** as the number of existing links divided by the number of possible links [39, 40, 41, 42]; the qualitative and quantitative connectance are calculated by dividing respectively the number of qualitative link density and quantitative link density by the number of species.

The **Nestedness** (NODF method) and the **Modularity** were calculated using R 3.0 software [43] using package ‘bipartite’ [44]. Because all parasitoids were found only a one leaf miner species, these two network descriptors were calculated only for the bi-partite networks composed by the plants and the leaf-miners. Food web graphics were drawn using R 3.0 software [43] using ‘Plotweb’ in package ‘bipartite’ [44].

## Results

### Amplification success

**Fig 1:**
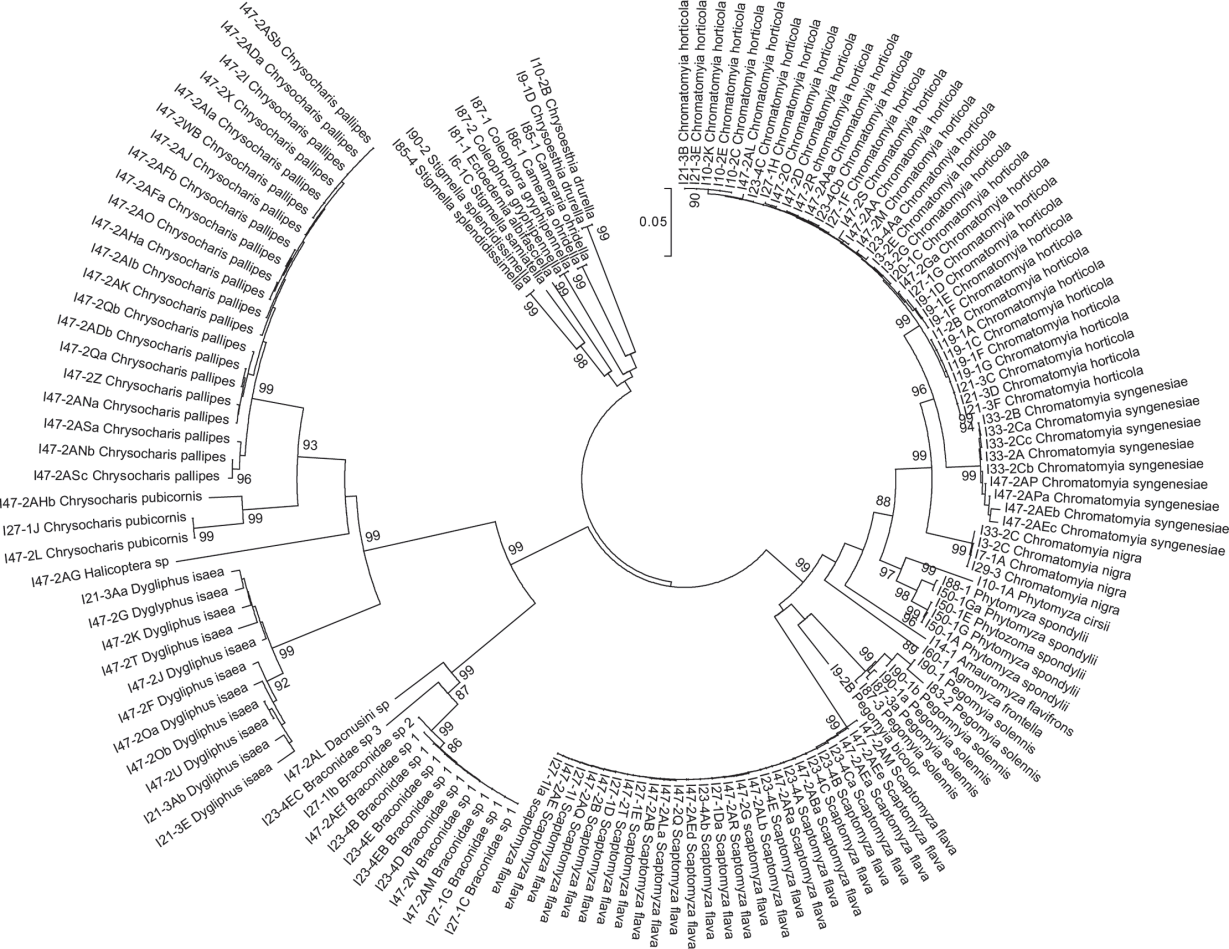
Maximum likelihood tree based on cytochrome c oxidase I (COI) Barcode sequences. Bootstrap values are indicated above branches. Scale bar indicates the number of substitutions per site.

**Fig 2:**
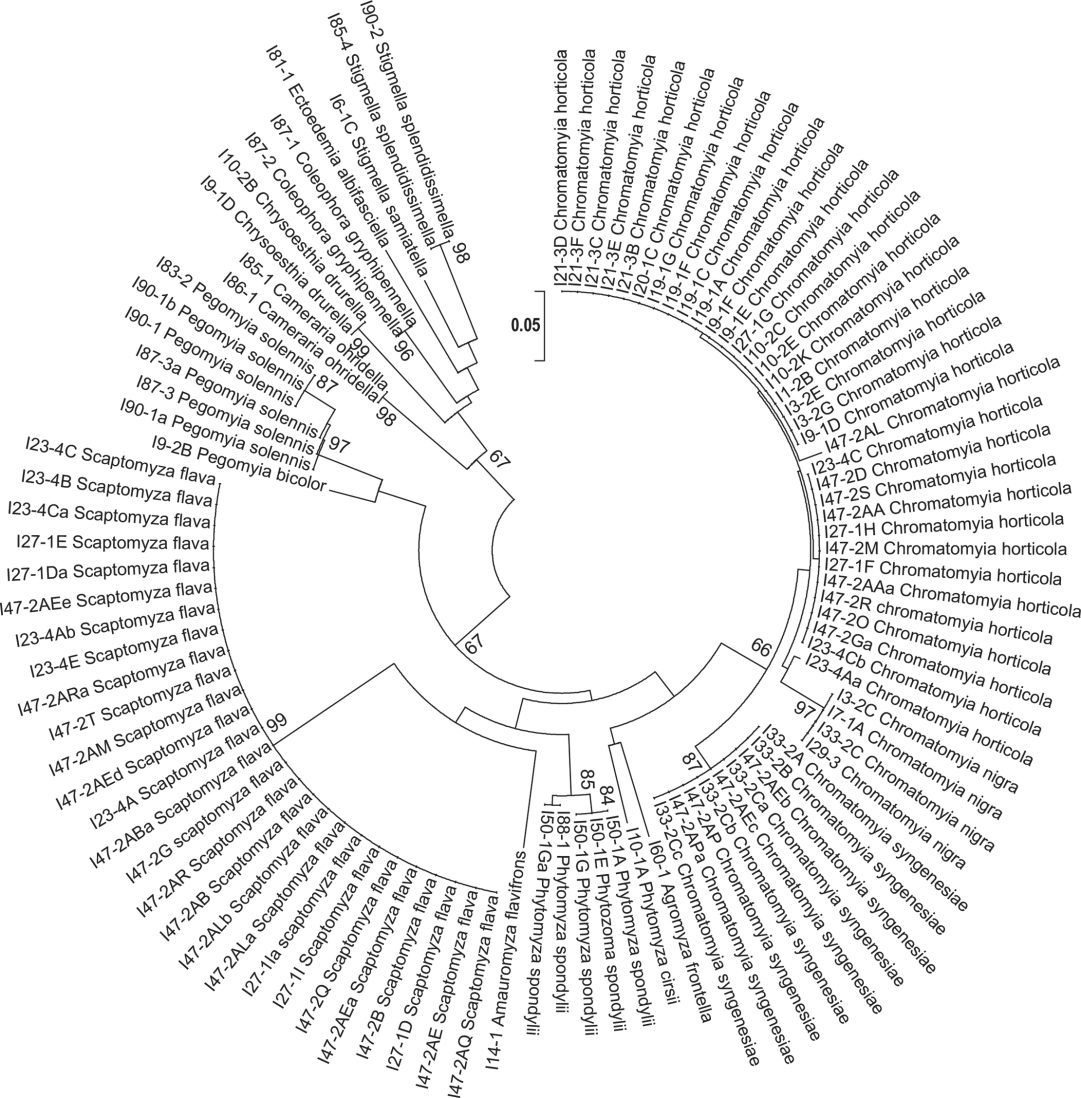
Maximum likelihood tree based on cytochrome c oxidase I (COI) Minibarcode sequences. Bootstrap values are indicated above branches. Scale bar indicates the number of substitutions per site.

A total of 147 insect specimens emerged or dissected from the 407 infested leaves collected: 89 dipteran leaf miners (68 adults and 21 immatures), 10 larvae of lepidopteran leaf miners and 48 adult hymenoptera parasitoids. The COI full barcode was successfully amplified from all adult and immature samples. The COI mini-barcode amplified with ZBJ-ArtF1c and ZBJ-ArtR2c primers was successfully amplified from all adult and immature Diptera and Lepidoptera species. The COI mini-barcode amplified with Uni-MinibarR1 and Uni-MinibarF1 primers was successfully amplified from all adult and immature Diptera and Lepidoptera species, but *Scaptomyza flava* (Fallén) and *Phytomyza spondylii* (Robineau-Desvoidy). The 658-bp sequences for COI full barcode (for all adult and immature specimens; Fig. 1) and the 130-bp sequences for COI mini-barocde (for all adults and immatures Diptera and Lepidoptera; Fig. 2) were used to assess nucleotide sequence divergence between and within morphological species and to perform the ML analysis. 260 infested leaves were empty: the leaf mining insects had already emerged when the leaves had been collected (Table 1).

**Table 1:** Number of leaves collected between the 13th June and the 19th September 2012.

	Infested leaves with insects ^a^	Infested leaves empty ^b^
*Aesculus hippocastanum*	2	4
*Chenopodim album*	2	3
*Cirsium arvense*	7	21
*Compositae* sp	1	11
*Epilobium* sp	1	3
*Heracleum sphondylium*	5	6
*Pisum sativum*	91	31
*Quercus robur*	1	21
*Quercus* sp	1	5
*Rosa canina*	2	7
*Rubus fruticosus*	2	23
*Rumex obtusifolius*	3	7
*Rumex* sp	2	13
*Stellaria graminea*	1	5
*Trifolium* sp	1	9
*Triticum aestivum*	25	91
Total	147	260

^a^ 147 leaves were still infested by a leaf mining insect when collected.

^b^ In 260 leaves, insects were already emerged when the leaves were collected.

For the amplification of the remaining DNA of mines (Diptera or Lepidoptera), the amplification success of the COI full barcode was very low (two mines representing 1.36% of the leaves analysed; Table 2). We found the best amplification success was with the ZBJ-ArtF1–ZBJ-ArtR2c primers (Table 2). We were able to amplify the remaining insect DNA from 48 infested leaves collected with an insect inside after emergence in the laboratory (147 leaves tested, success rate of 32.65%). We amplified the remaining insect DNA from 15 leaves collected with no insect inside the mine (i.e. 260 leaves tested, success rate of 5.77%; Table 2).

**Table 2:** Amplification success for the three primers pairs used on the infested leaves collected.

	Infested leaves^a^ (n = 147)	Empty leaves^b^ (n = 260)
LCO1490-HCO2198	2	0
Uni-MinibarF1 – Uni-MinibarR1	39	3
ZBJ-ArtF1 – ZBJ-ArtR2c	46	15
Total	48	15

^a^ Leaves were still infested by a leaf mining insect

^b^ No insect has been found within the infested leaves.

### Specimen identification

Of the 68 adult Diptera specimens, only 17 specimens could be identified to the species level based on the morphology of the adults (S1 Table). The identifications based on the shape of mine were unreliable, and showed numerous mismatches with both the adult identification and the molecular identification. For example, *S*. *flava*, *Chromatomyia horticola* (Gour) and *Chromatomyia syngenesiae* (Hardy) could not be reliably distinguished based on the shape their mines. In contrast, for Lepidoptera, the molecular identification and the identification based on the shape of the mines were consistent.

Concerning the molecular identification of adult specimens and immature stages dissected, the three methods of taxon assignment were consistent for the majority of the cases (S2 Table). All differences of identification were due to a sequence identity lower than 96% with megaBLAST. In these cases, the similarities found with BOLD animal identification were always over 99.45% and were consistent with the ML trees. Consequently, we based our molecular identification on these two methods.

**Fig 3:**
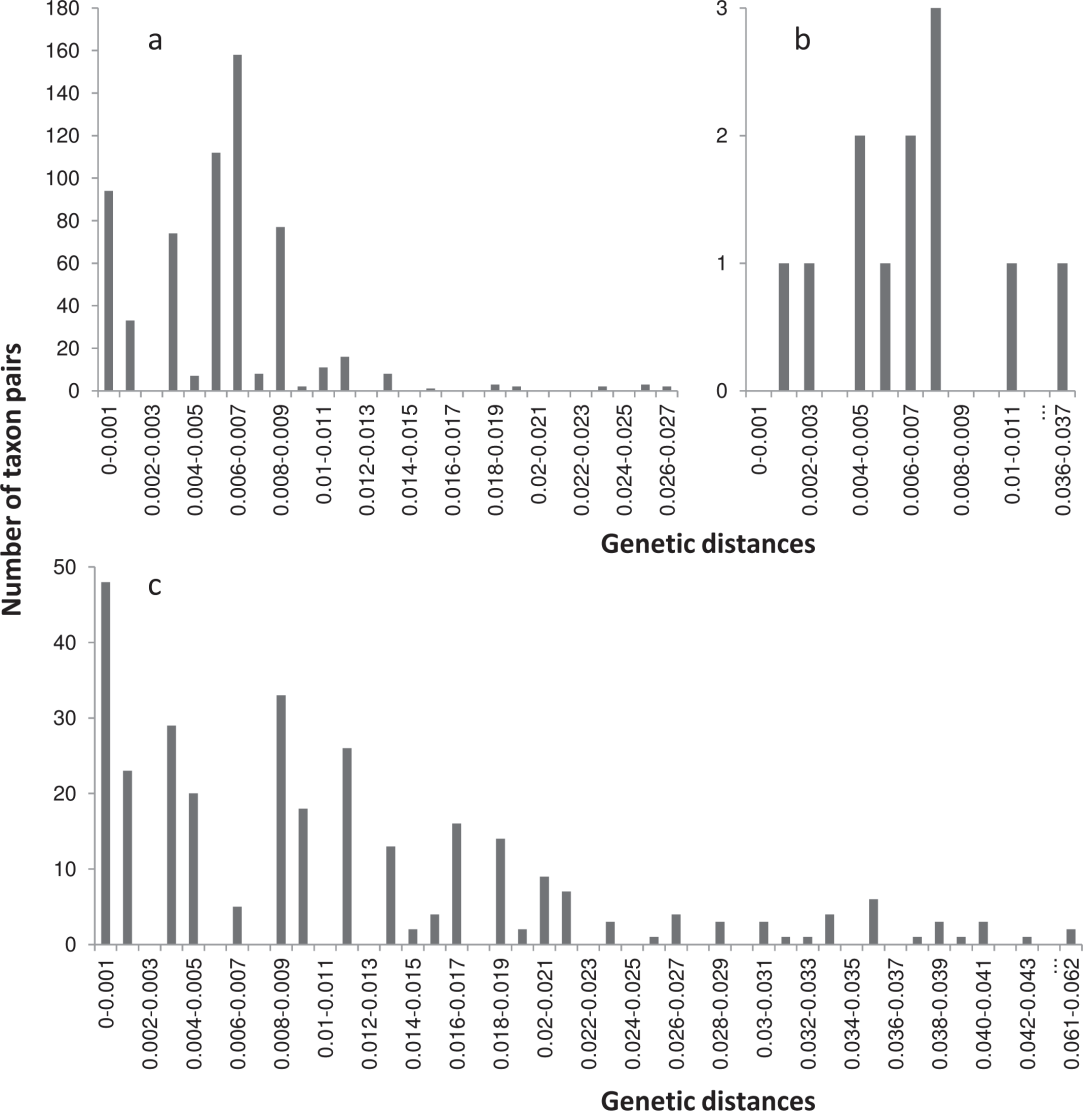
Intraspecific distance distribution for the DNA Barcode fragment of COI (658 bp). Intraspecific distance distribution for a) Agromyzidae species b) Anthomyiidae species c) Braconidae species for cytochrome c oxydase I (COI).

*Chromatomyia syngenesiae* COI full barcode sequences exhibited a high intraspecific variability (up to 0.028). *C*. *horticola*, *Pegomyia solennis* (Meigen) and *P*. *spondylii* (Robineau-Desvoidy) presented high intraspecific variability with both COI full barcode and minibarcode fragments (respectively 0.000 – 0.018 within *C*. *horticola*; 0.002 – 0.027 within *P*. *solennis* and 0.000 – 0.027 within *P*. *spondylii* for COI full barcode; 0.000 – 0.02 within *C*. *horticola*, *P*. *solennis* and *P*. *spondylii* for Minibarcode; Fig. 1; Fig. 2; Fig. 3). All Lepidoptera specimens were correctly identified by the COI full Barcode and Minibarcode (S1 Table; Fig. 1; Fig. 2).

*Diglyphus isaea* (Walker) was correctly identified with the COI full barcode and represents a very high intraspecific variability (between 0.000 and 0.041; Fig. 1; Fig. 3). Specimens identified morphologically as belonging to the genus *Chrysocharis* and *Halicoptera* were absent from Genbank and BOLD databases and therefore cannot be identified based on their COI sequences. Finally, we were unable to identify twelve Braconidae specimens to species-level with both morphological and molecular identification. However, COI full barcode sequences distinguished four different clusters in the ML tree, one of them identified as *Dacnusa* sp. (Fig. 1; interspecific variability: 0.005–0.1004).

We were able to identify to species level all DNA detected from the leaf mines. These molecular identifications are reported in S1 Table for the leaves where an insect (adult or immature) was found. Fifteen samples from leaves that were empty upon collection were successfully amplified and identified (Table 2): we found 1 *Phytomyza cirsii* (Hendel) and 1 *C*. *syngenesiae* on *Cirsium arvense* (L.); 2 *C*. *horticola* and 1 *Chromatomyia* sp on *Pisum sativum* (L.); 3 *Stigmella samiatella* (Zeller) and 2 *Ectoedemia albifasciella* (Heinemann) on *Quercus robur* (L.); 1 *Stigmella splendidissimella* (Herrich-Schaffer) on *Rubus fruticosus* (L.); 4 *C*. *horticola* on *Triticum aestivum* (L.). Overall, we were able to identify leaf miner species (Diptera or Lepidoptera) but not parasitoid species (Hymenoptera) using the remaining DNA within the leaf mines.

### Ecological network of plants – leaf mining insects – parasitoids

**Fig 4:**
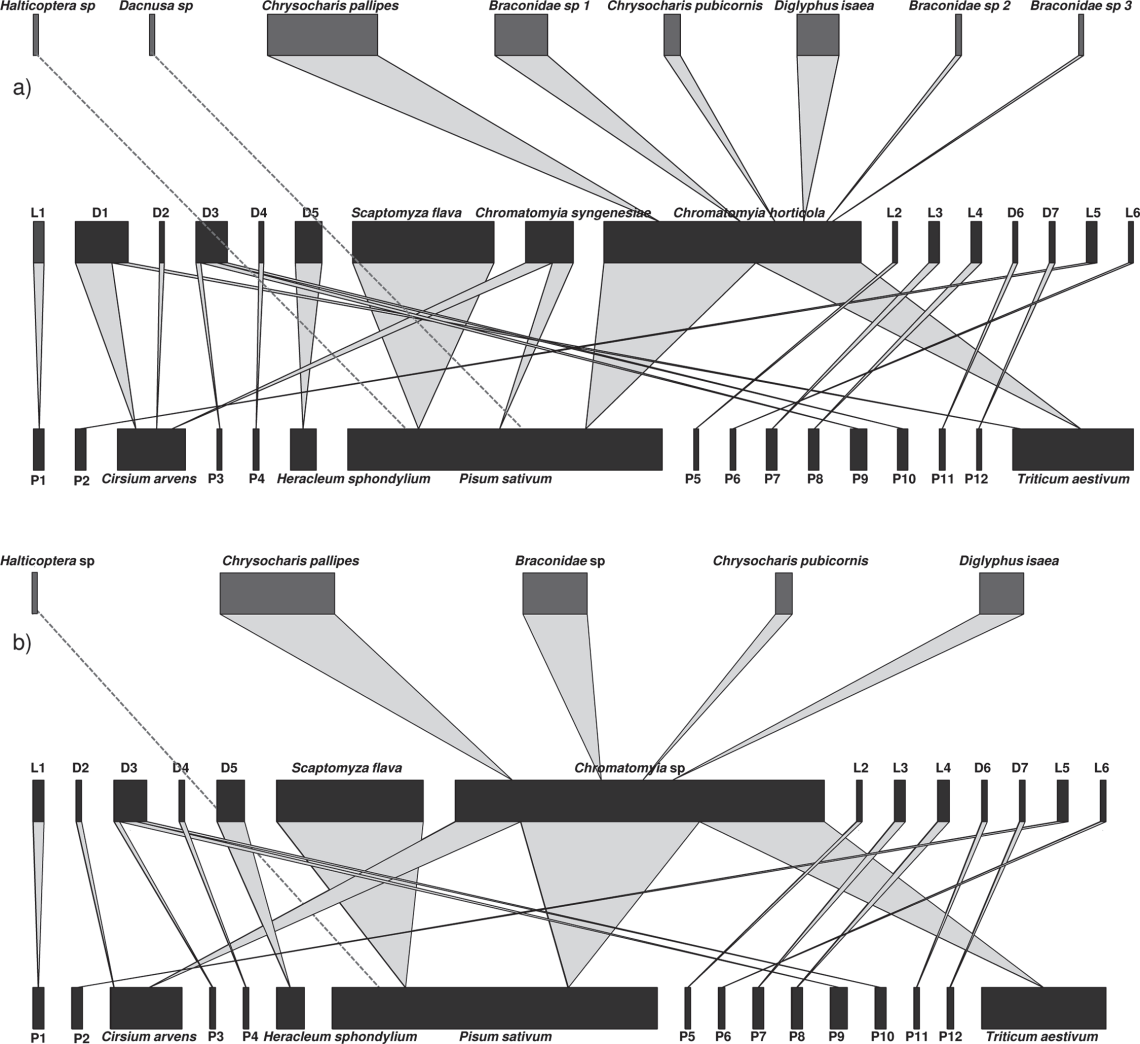
Quantitative food webs between plants, leaf mining insects and their parasitoids. The food webs were constructed from 407 infested leaves sampled at Stockbridge Technology Center (Cawood, United Kingdom) using a) a molecular approach b) a morphological identification. The series of bars represent plant abundance (bottom), leaf mining insect abundance (middle) and parasitoid abundance (top). The width of edge links between plants, leaf-miners and parasitoids illustrates the relative strength of each interaction. Dashed lines are used where we are unable to identify the insect host of a parasitoid. L1: *Cameraria orhidella*; L2: *Stigmella samiatella*; L3: *Coleophora gryphipennella*; L4: *Stigmella splendidissimella*; L5: *Chrysoesthia drurella*; L6: *Ectoedemia albifasciella*; D1: *Chromatomyia nigra*; D2: *Phytomyza cirsii*; D3: *Pegomyia solennis*; D4: *Pegomyia bicolor*; D5: *Phytomyza spondylii*; D6: *Amauromyza flavifrons*; D7: *Agromyza frontella*; P1: *Aesculus hippocastanum*; P2: *Chenopodium album*; P3: *Compositae sp*; P4: *Epilobium sp*; P5: *Quercus robur*; P6: *Quercus sp*; P7: *Rosa canina*; P8: *Rubus fruticosus*; P9: *Rumex obtusifolius*; P10: *Rumex sp*; P11: *Stellaria graminea*; P12: *Trifolium sp*.

We built an ecological network consisting of plants – leaf mining insects – parasitoids using the molecular identification of insect specimens with the COI full barcode and the remaining DNA of leaf mining insects within the mines with the COI Minibarcode (Fig. 4A). When a parasitoid emerged from an infested leaf and we successfully detected and identified the leaf mining insect based on the remaining DNA of the mine, we were able to determine a tripartite interaction: we were able to find the leaf miner host for 15 of the 48 parasitoids emerged. In all other cases, we describe the bipartite interaction between 1) plants and leaf mining insects 2) plants and leaf miner parasitoids. We consider in the molecular network the four different Braconidae clusters found with COI ML tree as four different species due to the high interspecific variability (0.005–0.1004).

We compared this molecular network to an ecological network of plants – leaf mining insects – parasitoids based on morphological indentification of adult specimens and mines (only when adult specimens were not available; Fig. 4B). In the morphological network, *C*. *horticola*, *C*. *nigra* and *C*. *syngenesiae* are considered as a single species because it was not possible to distinguish between these three leaf miner species using morphological criteria. The four different Braconidae clusters (*Braconidae* sp. 1, *Braconidae* sp. 2, *Braconidae* sp. 3 and *Dacnusa* sp.) were considered as a single species because we could not distinguish between these four parasitoid species with morphological criteria.

The network constructed using the molecular approach had a higher number of species, number of links, qualitative link density, qualitative vulnerability and plant–leaf miner modularity compared to the network constructed based on morphological characteristics (Table 3). However, the quantitative link density, the quantitative connectance and the plant–leaf miner nestedness are lower in molecular network than in morphological network. Finally, the measures of qualitative generality and the qualitative connectance were consistent between the network types.

**Table 3:** Comparison of molecular food web and morphological food web structure.

	Molecular network	Morphological network
Number of species	40	35
Number of links	29	23
Qualitative Link density	0.725	0.657
Quantitative Link densisty	2.075	2.371
Qualitative Connectance	0.018	0.019
Quantitative Connectance	0.052	0.068
Qualitative Vulnerability	0.906	0.767
Quantitative Vulnerability	0.692	0.692
Qualitative Generality	1.208	1.211
Quantitative Generality	1.446	1.446
Plant Leaf-miner NODF	0.833	0.948
Plant Leaf-miner Modularity	0.485	0.434

## Discussion

We provide a novel and more accurate methodology for constructing plant – leaf miner – parasitoid networks. Moreover, we show how a molecular approach can be used to determine difficult and cryptic species interactions, even when an adult insect has left its leaf mine. Our molecular approach found more species and interactions than traditional approaches based on insect rearing methods, altering network structure as well as identifying previously unknown species interactions. Thus networks constructed using these molecular methods are better resolved and more useful for network ecologists.

### Molecular and morphological identification

A dissection of the male genitalia to identify the leaf mining Diptera [22] involved in this study was performed to identify specimens to species level, which precludes the inclusion of females and immature specimens. Without a molecular approach, which has no such age or gender restrictions, more than 50% of specimens collected during our field season would have been impossible to include in the ecological network analysis. None of our Lepidopteran specimens emerged as adults, but we could overcome this problem using a molecular approach and we recovered ten full barcode sequences from immature Lepidoptera to include in the network. DNA full barcode sequences allowed us to detect and identify four separate morphologically cryptic Braconidae species and 12 morphologically identitical parasitoids associated with them.

### Methodological considerations

One of the most significant challenges to identifying leaf miners and their parasites is the small number of sequences present in the public sequence databases. Consequently, some specimens are not identified to the species-level with molecular markers because we were not able to find any matching sequences in the databases. This was the case for the Diptera *Pegomya solennis* and *Pegomya bicolour* and for the Hymenoptera *Chrysocharis pubicornis* and *Chrysocharis pallipes*, which were identified by their morphology. Despite the lack of a reference sequence in the public databases however the phylogenetic analysis of barcode sequences can often identify the phylogenetic position of the unknown sample, allowing taxonomic inference and further directed research. This was the case with twelve Braconidae specimens: they were morphologically identical but using COI full barcode sequences we distinguished four different Braconidae clusters. These Braconidae clusters need further examination, more specimens and sequences data to determine whether or not they are different species.

One of the main challenges for efficient molecular identification is to greatly extend the database of barcode sequences and voucher specimens [45]. Studies of phylogeny have considerably increased the completion of databases for some leaf miner groups by sequencing the COI full barcode fragment (e.g [46] for the Agromyzidae family; [47] for the Coleophoridae). One of the next steps of our approach is to extend the database for the other leaf miner groups and their parasitoids.

### Improving the success rate of identifying leaf mining insects from DNA within mines

Our study demonstrates that a 130 bp COI minibarcode sequence [27, 28] is enough to identify all specimens to the species level included in this study where we have reference sequences available. The minibarcode can be used to identify the leaf miners using DNA remaining in the mines, even where that DNA is likely degraded. It appears to be far more accurate than using the shape of the mine to identify the phytophagous insect, at least for the Diptera species. Although the identification based on the DNA and the shapes of the mines are fully consistent for the Lepidoptera species, we found a lot of mismatches for the Diptera species. The most common error found was confusion between *Chromatomyia horticola* and *Scaptomyza flava*, even if these species are quite distant in a phylogenetic context. This highlights the advantage of using a molecular approach, like DNA barcoding, that places the unknown taxon into an evolutionary framework (phylogeny), rather than morphological approaches like mine shape, that can provide no information outside of their character matching.

The amplification of the DNA from the mines provides important ecological information, even if some improvements in the rate of amplification are still required. Overall, we were able to amplify more than 16% of the infested leaves collected. The rate increased to 33% if we consider only the samples where the insect emerged in the laboratory from the leaf mine. However, we were able to amplify only 6% of the infested leaves that were empty when collected in the field. This is perhaps not so surprising as it was not possible to determine the time since emergence in field-collected leaves and some may be weeks or months old with little remaining DNA. More research is necessary to determine how long after the emergence of an insect it is still possible to routinely amplify DNA within the mine. This answer is complicated by the fact that the threshold will likely depend on both insects and plants, but conducting an extensive rearing programme should provide this key information. It was clear during our study that the degradation of pea and wheat leaves was far faster than the leaves collected from the trees. Even if a short fragment can assist in the amplification of degraded DNA [26, 27, 28, 45], bad conditions such as high humidity and incident light are likely to degrade the DNA so that it is no longer amplifiable using standard conditions. We did not, in this experiment, optimise PCR conditions for retrieval and amplification of highly degraded DNA from leaf mines, which will be a fruitful future direction. We think nevertheless that the primers we used, especially ZBJ-ArtF1c ZBJ-ArtR1c, were perfectly adequate for the purpose of this study (i.e to amplify the degraded DNA of lepidopteran and dipteran leafminers). Indeed, several studies have demonstrated that these mini-barcode primers are very good to amplify Lepidoptera and Diptera degraded DNA [27], [48], [49]. We have clearly shown however that even without specific ‘ancient DNA’ measures we can retrieve considerable information for our ecological networks from DNA remaining in mines. Consequently, even if improvements in the method are needed, our molecular approach adds adequately to the other existing tools (i.e. insect morphology, the mine shape) and increase the accuracy of the interactions described in ecological networks. The present study therefore opens up the use of degraded DNA from leaf mines to describe plant–leaf miner interactions.

### Cryptic species in leaf mining insects and their parasitoids

Several species in our study show a high and phylogenetically structured intraspecific variability, which often indicates the presence of cryptic species. These include the Hymenoptera *Dygliphus isaea* and in a less extend the leaf miners *Chromatomyia horticola*, *Chromatomyia syngenesiae*, *Pegomya solennis*, *Phytomyza sphondylii*. Our results therefore support the hypothesis of Sha et al. [50] in considering *D*. *isaea* as a complex of species; however, to our knowledge, it is the first time that a possible complex of species has been suggested for the other species. Further sampling of these species needs to be conducted to confirm and characterise the host range of these potentially cryptic species. A more exhaustive study of the variability of molecular markers found in the leaf miners and their parasitoids would be informative as it would demonstrate whether this high intraspecific variability is common or not among these organisms and then lead to a rigourous analysis of cryptic species [51].

The presence of cryptic species can modify the structure of an ecological network [14], especially in the case of plant – leaf miner – parasitoid food-webs as cryptic species have been found in the second and third trophic levels. Consequently, it is necessary to be very careful when we name the species and describe the interactions in an ecological network as they can be biased by the existence of cryptic specialists.

### Ecological networks of plants – leaf miners – parasitoids and new molecular tools to improve our knowledge

Our molecular approach allowed us to build a highly resolved tripartite ecological network. While this ecological network is far from being exhaustive, and more sampling is needed, we were nevertheless able to find previously unknown interactions including the tripartite interaction between *Pisum sativum*, *Chromatomyia horticola* and *Chrysocharis pallipes*, and the bipartite interaction between *Epilobium sp* and *Pegomya bicolor*. There is clearly a lack of basic knowledge about plant – leafminer – parasitoid interactions in farmland ecosystems, despite their importance for agriculture, and DNA barcoding approaches will be indispensable to resolve interactions in these ecological networks [52]. Indeed, our study shows that using this molecular approach affects network structure compared to traditional approaches, with implications for network-level analysis. We demonstrate that our molecular approach affects qualitative network metrics such as the number of species, the number of links and vulnerability compared to morphological network descriptors and that it also affects quantitative network metrics such as the link density, connectance, nestedness and the modularity of plant–leaf miner interactions. However, because it is likely that we did not sample the complete range of species-interactions, these network descriptors need to be used with caution, especially if they are integrated into a wider analysis (i.e. meta-analysis of tri-partite ecological networks).

The leaf miners and the parasitoids in this study are essentially attacking one (or a very few) hosts and the low number of links in the morphological network can be explained by a lower number of leaf miners and parasitoids identified. The differences in topology between the molecular and morphogical ecological networks could be greater if we consider as different species the clusters based on COI sequences within the following morpho-groups: the leaf miners *C*.*syngenesiae*, *C*. *horticola*, *P*. *solennis*, *P*. *spondylii* and the parasitoids *D*. *isaea* and *C*. *pubicornis*.

We suggest our method should be used to complement molecular approaches developed by Wirta et al. [18]. They succeeded in developing a molecular tool that was able to detect and identify parasitoid immature stages within leaf miner larvae with parasitoid group-specific primer pairs. Moreover, they were able to identify the host DNA within the gut contents of new emerged parasitoids. Consequently, a complementary use of all of these molecular tools will considerably improve our capacity to build highly resolved ecological networks. Such perspectives will lead to new and exciting research opportunities in community ecology as well as applied biological control.

Understanding plant – leaf miner – parasitoid interactions and farmland ecological networks more generally is necessary because natural pest control is becoming a major issue in crop production and food security [53, 54]. Our molecular approach provides a novel way of better understanding difficult to observe species interactions within ecological networks. Thus, a combination of novel molecular approaches and ecological network analysis is likely to enable agro-ecologists to identify the species of agronomic interest and to find ways of enhancing natural pest control.

## Data Accessibility

DNA sequences: COI sequences were assigned GenBank accessions: KM073108 to KM073254.

Sequence alignements and plant–host–parasitoid networks were assigned Dryad doi:10.5061/dryad.5dr5k.

## Supporting Information

S1 Table List of species included in this study. Voucher number (D: Diptera; L: Lepidoptera; H: Hymenoptera), plant host species, identification based on the leaf mine, morphological identification of the adult specimen, molecular identification base on COI barcode fragment. We specify which primer pairs were able to detect and identify the leaf mining insects using the remaining DNA within the mines (LCO: LCO1490-HCO2198; ZBJ: ZBJ-ArtF1–ZBJ-ArtR2c; Mini: Uni-MinibarF1-Uni-MinibarR1).
(DOCX)

S2 Table Comparison of three methods of molecular identification: BOLD similarity, Genbank megaBLAST and Maximum Likelihood Tree. (DOCX)
