# Supplementary material for: Determining Plant – Leaf Miner – Parasitoid Interactions: A DNA Barcoding Approach
Source: PLoS One. 2015 Feb 24;10(2):e0117872. doi: 10.1371/journal.pone.0117872 (PMC4339730; doi:10.1371/journal.pone.0117872)
Supplement: S1 Table — Voucher number (D: Diptera; L: Lepidoptera; H: Hymenoptera), plant host species, identification based on the leaf mine, morphological identification of the adult specimen, molecular identification base on COI barcode fragment. We specify which primer pairs were able to detect and identify the leaf mining insects using the remaining DNA within the mines (LCO: LCO1490-HCO2198; ZBJ: ZBJ-ArtF1–ZBJ-ArtR2c; Mini: Uni-MinibarF1-Uni-MinibarR1). (DOCX) [file pone.0117872.s001.docx]

**Supporting Information**

**Table S1**. **List of species included in this study.**

| Voucher number | Host plant | Shape of the trail | Insect morphology | COI barcode | Amplification of the trail | Minibarcode trail |
| --- | --- | --- | --- | --- | --- | --- |
| L-I86-1 | A. hippocastanum | Cameraria ohridella | | Cameraria ohridella | Mini, ZBJ | Cameraria ohridella |
| L-I85_1 | A. hippocastanum | Cameraria ohridella | | Cameraria ohridella | 0 |  |
| L-I10-2B | C. album | Chrysoesthia drurella | | Chrysoesthia drurella | 0 |  |
| L-I9-1D | C. album | Chrysoesthia drurella | | Chrysoesthia drurella | 0 |  |
| D-I10-1A | C. arvense | Phytomyza cirsii |  | Phytomyza cirsii | Mini, ZBJ | Phytomyza cirsii |
| D-I33-2A | C. arvense | Chromatomyia 'atricornis' | Chromatomyia sp. | Chromatomyia syngenesiae | Mini | Chromatomyia syngenesiae |
| D-I33-2B | C. arvense | Chromatomyia 'atricornis' | Chromatomyia sp. | Chromatomyia syngenesiae | 0 |  |
| D-I33-2C | C. arvense | Chromatomyia 'atricornis' | Chromatomyia horticola | Chromatomyia nigra | 0 |  |
| D-I33-2Ca | C. arvense | Chromatomyia 'atricornis' | Chromatomyia horticola | Chromatomyia syngenesiae | Mini, ZBJ | Chromatomyia syngenesiae |
| D-I33-2Cb | C. arvense | Chromatomyia 'atricornis' | Chromatomyia sp. | Chromatomyia syngenesiae | 0 |  |
| D-I33-2Cc | C. arvense | Chromatomyia 'atricornis' | Chromatomyia sp. | Chromatomyia syngenesiae | Mini, ZBJ | Chromatomyia syngenesiae |
| D-I83-2 | Compositae sp |  | Pegomyia solennis | Pegomya solennis | 0 |  |
| D-I9-2B | Epilobium sp. |  | Pegomyia bicolor | Pegomyia bicolor | ZBJ | Anthomyiidae sp. |
| D-I50-1A | H. sphondylium | Phytomyza spondylii | Phytomyza spondylii | Phytomyza spondylii | 0 |  |
| D-I50-1E | H. sphondylium | Phytomyza spondylii | Phytomyza spondylii | Phytomyza spondylii | 0 |  |
| D-I50-1G | H. sphondylium | Phytomyza spondylii | Chromatomyia sp. | Phytomyza spondylii | 0 |  |
| D-I50-1Ga | H. sphondylium | Phytomyza spondylii | Chromatomyia sp. | Phytomyza spondylii | 0 |  |
| D-I88-1 | H. sphondylium | Phytomyza spondylii | | Phytomyza spondylii | ZBJ | Phytomyza spondylii |
| D-I23-4A | P. sativum | Chromatomyia horticola | Scaptomyza sp. | Scaptomyza flava | 0 |  |
| D-I23-4Aa | P. sativum | Chromatomyia horticola | Chromatomyia sp. | Chromatomyia horticola | 0 |  |
| D-I23-4Ab | P. sativum | Chromatomyia horticola | Chromatomyia sp. | Chromatomyia horticola | 0 |  |
| D-I23-4AE | P. sativum | Chromatomyia horticola | Scaptomyza flava | Scaptomyza flava | 0 |  |
| D-I23-4B | P. sativum | Chromatomyia horticola | Scaptomyza sp. | Scaptomyza flava | 0 |  |
| D-I23-4C | P. sativum | Chromatomyia horticola | Scaptomyza sp. | Chromatomyia horticola | Mini, ZBJ | Chromatomyia horticola |
| D-I23-4Ca | P. sativum | Chromatomyia horticola | Scaptomyza sp. | Scaptomyza flava | 0 |  |
| D-I23-4Cb | P. sativum | Chromatomyia horticola | Chromatomyia sp. | Chromatomyia horticola | Mini, ZBJ | Chromatomyia horticola |
| D-I23-4Cc | P. sativum | Chromatomyia horticola | Scaptomyza sp. | Scaptomyza flava | ZBJ | Scaptomyza flava |
| D-I27-1D | P. sativum | Scaptomyza flava | Scaptomyza sp. | Scaptomyza flava | 0 |  |
| D-I27-1Da | P. sativum | Scaptomyza flava | Scaptomyza sp. | Scaptomyza flava | 0 |  |
| D-I27-1E | P. sativum | Scaptomyza flava | Scaptomyza sp. | Scaptomyza flava | 0 |  |
| D-I27-1F | P. sativum | Scaptomyza flava | Chromatomyia horticola | Chromatomyia horticola | Mini, ZBJ | Chromatomyia horticola |
| D-I27-1G | P. sativum | Scaptomyza flava | Chromatomyia sp. | Chromatomyia horticola | 0 |  |
| D-I47-2AA | P. sativum | Scaptomyza flava | Chromatomyia sp. | Chromatomyia horticola | Mini, ZBJ, LCO | Chromatomyia horticola |
| D-I47-2AAa | P. sativum | Scaptomyza flava | Chromatomyia sp. | Chromatomyia horticola | 0 |  |
| D-I47-2AB | P. sativum | Scaptomyza flava | Scaptomyza sp. | Scaptomyza flava | ZBJ | Scaptomyza flava |
| D-I47-2ABa | P. sativum | Scaptomyza flava | Scaptomyza sp. | Scaptomyza flava | 0 |  |
| D-I47-2AE | P. sativum | Scaptomyza flava | Scaptomyza flava | Scaptomyza flava | 0 |  |
| D-I47-2AEa | P. sativum | Scaptomyza flava | Scaptomyza sp. | Scaptomyza flava | 0 |  |
| D-I47-2AEe | P. sativum | Scaptomyza flava | Scaptomyza sp. | Scaptomyza flava | 0 |  |
| D-I47-2AL | P. sativum | Scaptomyza flava | Chromatomyia sp. | Chromatomyia horticola | 0 |  |
| D-I47-2ALa | P. sativum | Scaptomyza flava | Scaptomyza sp. | Scaptomyza flava | 0 |  |
| D-I47-2ALb | P. sativum | Scaptomyza flava | Scaptomyza sp. | Scaptomyza flava | 0 |  |
| D-I47-2AP | P. sativum | Scaptomyza flava | Chromatomyia sp. | Chromatomyia syngenesiae | 0 |  |
| D-I47-2APa | P. sativum | Scaptomyza flava | Chromatomyia sp. | Chromatomyia syngenesiae | ZBJ | Chromatomyia syngenesiae |
| D-I47-2AQ | P. sativum | Scaptomyza flava | Scaptomyza flava | Scaptomyza flava | ZBJ | Scaptomyza flava |
| D-I47-2AR | P. sativum | Scaptomyza flava | Scaptomyza flava | Scaptomyza flava | 0 |  |
| D-I47-2ARa | P. sativum | Scaptomyza flava | Scaptomyza flava | Scaptomyza flava | 0 |  |
| D-I47-2B | P. sativum | Scaptomyza flava | Scaptomyza sp. | Scaptomyza flava | 0 |  |
| D-I47-2D | P. sativum | Scaptomyza flava | Chromatomyia horticola | Chromatomyia horticola | Mini, ZBJ | Chromatomyia horticola |
| D-I47-2G | P. sativum | Scaptomyza flava | Scaptomyza sp. | Scaptomyza flava | 0 |  |
| D-I47-2Ga | P. sativum | Scaptomyza flava | Scaptomyza sp. | Scaptomyza flava | ZBJ | Scaptomyza flava |
| D-I47-2M | P. sativum | Scaptomyza flava | Chromatomyia sp. | Chromatomyia horticola | 0 |  |
| D-I47-2O | P. sativum | Scaptomyza flava | Chromatomyia horticola | Chromatomyia horticola | 0 |  |
| D-I47-2R | P. sativum | Scaptomyza flava | Chromatomyia sp. | Chromatomyia horticola | 0 |  |
| D-I47-2T | P. sativum | Scaptomyza flava | Scaptomyza sp. | Scaptomyza flava | 0 |  |
| D-I27-1H | P. sativum | Scaptomyza flava | | Chromatomyia horticola | Mini, ZBJ | Chromatomyia horticola |
| D-I27-1I | P. sativum | Scaptomyza flava | | Scaptomyza flava | 0 |  |
| D-I27-1Ia | P. sativum | Scaptomyza flava | | Scaptomyza flava | 0 |  |
| D-I47-2AEb | P. sativum | Scaptomyza flava | | Chromatomyia syngenesiae | Mini, ZBJ | Chromatomyia syngenesiae |
| D-I47-2AEc | P. sativum | Scaptomyza flava | | Chromatomyia syngenesiae | 0 |  |
| D-I47-2AEd | P. sativum | Scaptomyza flava | | Scaptomyza flava | ZBJ | Scaptomyza flava |
| D-I47-2AM | P. sativum | Scaptomyza flava | | Scaptomyza flava | 0 |  |
| D-I47-2Q | P. sativum | Scaptomyza flava | | Scaptomyza flava | 0 |  |
| D-I47-2S | P. sativum | Scaptomyza flava | | Chromatomyia horticola | 0 |  |
| L-I6_1C | Q. robur | Stigmella samiatella | | Stigmella samiatella | Mini, ZBJ | Stigmella samiatella |
| L-I81-1 | Quercus sp. | Ectoedemia sp. |  | Ectoedemia albifasciella | Mini, ZBJ | Eectoedemia albifasciella |
| L-I87-1 | R. canina | Coleophora gryphipennella | | Coleophora gryphipennella | Mini, ZBJ | Coleophora gryphipennella |
| L-I87-2 | R. canina | Coleophora gryphipennella | | Coleophora gryphipennella | 0 |  |
| L-I90-2 | R. fruticosus | Stigmella splendidissimella | | Stigmella splendidissimella | 0 |  |
| L-I85_4 | R. fruticosus | Stigmella splendidissimella | | Stigmella splendidissimella | Mini, ZBJ | Stigmella splendidissimella |
| D-I90-1 | R. obtusifolius | Pegomya solennis | | Pegomya solennis | Mini, ZBJ | Diptera sp. |
| D-I90-1a | R. obtusifolius | Pegomya solennis | | Pegomya solennis | 0 |  |
| D-I90-1b | R. obtusifolius | Pegomya solennis | | Pegomya solennis | 0 |  |
| D-I87-3 | Rumex sp. | Pegomya solennis | | Pegomya solennis | Mini, ZBJ | Diptera sp. |
| D-I87-3a | Rumex sp. | Pegomya solennis | | Pegomya solennis | Mini, ZBJ | Diptera sp. |
| D-I14-1 | S. graminea |  |  | Amauromyza flavifrons | 0 |  |
| D-I10-2C | T. aestivum | Chromatomyia 'atricornis' | Chromatomyia sp. | Chromatomyia horticola | 0 |  |
| D-I10-2E | T. aestivum | Chromatomyia 'atricornis' | Chromatomyia sp. | Chromatomyia horticola | 0 |  |
| D-I10-2K | T. aestivum | Chromatomyia 'atricornis' | | Chromatomyia horticola | 0 |  |
| D-I1-2B | T. aestivum | Chromatomyia 'atricornis' | Chromatomyia sp. | Chromatomyia horticola | 0 |  |
| D-I19-1G | T. aestivum |  | Chromatomyia horticola | Chromatomyia horticola | Mini, ZBJ | Chromatomyia horticola |
| D-I21-3C | T. aestivum | Chromatomyia 'atricornis' | Chromatomyia sp. | Chromatomyia horticola | 0 |  |
| D-I21-3D | T. aestivum | Chromatomyia 'atricornis' | Chromatomyia sp. | Chromatomyia horticola | 0 |  |
| D-I21-3E | T. aestivum | Chromatomyia 'atricornis' | Chromatomyia horticola | Chromatomyia horticola | 0 |  |
| D-I21-3F | T. aestivum | Chromatomyia 'atricornis' | Chromatomyia sp. | Chromatomyia horticola | 0 |  |
| D-I29-3 | T. aestivum |  | Chromatomyia sp. | Chromatomyia nigra | 0 |  |
| D-I3-2C | T. aestivum | Agromyza sp. | Chromatomyia sp. | Chromatomyia nigra | 0 |  |
| D-I3-2E | T. aestivum | Chromatomyia 'atricornis' | Chromatomyia sp. | Chromatomyia horticola | 0 |  |
| D-I3-2G | T. aestivum | Chromatomyia 'atricornis' | Chromatomyia sp. | Chromatomyia horticola | 0 |  |
| D-I7-1A | T. aestivum | Agromyza sp. | Chromatomyia sp. | Chromatomyia nigra | Mini, ZBJ | Chromatomyia nigra |
| D-I9-1D | T. aestivum | Chromatomyia 'atricornis' | Chromatomyia sp. | Chromatomyia horticola | 0 |  |
| D-I9-1E | T. aestivum | Chromatomyia 'atricornis' | Chromatomyia horticola | Chromatomyia horticola | 0 |  |
| D-I9-1F | T. aestivum | Chromatomyia 'atricornis' | Chromatomyia sp. | Chromatomyia horticola | 0 |  |
| D-I19-1A | T. aestivum |  |  | Chromatomyia horticola | Mini,ZBJ,LCO | Chromatomyia horticola |
| D-I19-1C | T. aestivum |  | Chromatomyia sp. | Chromatomyia horticola | Mini, ZBJ | Chromatomyia horticola |
| D-I19-1F | T. aestivum |  | Chromatomyia horticola | Chromatomyia horticola | 0 |  |
| D-I20-1C | T. aestivum |  |  | Chromatomyia horticola | Mini, ZBJ | Chromatomyia horticola |
| D-I21-3B | T. aestivum | Chromatomyia 'atricornis' | | Chromatomyia horticola | ZBJ | Chromatomyia horticola |
| D-I60-1 | Trifolium sp. | Agromyza frontella | | Agromyza frontella | 0 |  |
| H-I23-4B | P. sativum | Chromatomyia horticola | Braconidae sp. | Braconidae sp. | 0 |  |
| H-I23-4D | P. sativum | Chromatomyia horticola | Braconidae sp. | Braconidae sp. | 0 |  |
| H-I23-4E | P. sativum | Chromatomyia horticola | Braconidae sp. | Braconidae sp. | Mini, ZBJ | Chromatomyia horticola |
| H-I23-4EB | P. sativum | Scaptomyza flava | Braconidae sp. | Braconidae sp. | 0 |  |
| H-I27-1C | P. sativum | Scaptomyza flava | Braconidae sp. | Braconidae sp. | Mini, ZBJ | Chromatomyia horticola |
| H-I27-1G | P. sativum | Scaptomyza flava | Braconidae sp. | Braconidae sp. | 0 |  |
| H-I27-1Ib | P. sativum | Scaptomyza flava | Braconidae sp. | Braconidae sp. | Mini, ZBJ | Chromatomyia horticola |
| H-I47-2AEf | P. sativum | Scaptomyza flava | Braconidae sp. | Braconidae sp. | Mini, ZBJ | Chromatomyia horticola |
| H-I47-2AM | P. sativum | Scaptomyza flava | Braconidae sp. | Braconidae sp. | 0 |  |
| H-I47-2W | P. sativum | Scaptomyza flava | Braconidae sp. | Braconidae sp. | 0 |  |
| H-I23-4EC | P. sativum | Scaptomyza flava | Braconidae sp. | Braconidae sp. | 0 |  |
| H-I47-2AL | P. sativum | Scaptomyza flava | Braconidae sp. | Dacnusa sp. | 0 |  |
| H-I21-3Aa | T. aestivum | Chromatomyia 'atricornis' | Diglyphus isaea | Diglyphus isaea | 0 |  |
| H-I21-3Ab | T. aestivum | Chromatomyia 'atricornis' | Diglyphus isaea | Diglyphus isaea | 0 |  |
| H-I21-3E | T. aestivum | Chromatomyia 'atricornis' | Diglyphus isaea | Diglyphus isaea | Mini, ZBJ | Chromatomyia horticola |
| H-I47-2F | P. sativum | Scaptomyza flava | Diglyphus isaea | Diglyphus isaea | 0 |  |
| H-I47-2G | P. sativum | Scaptomyza flava | Diglyphus isaea | Diglyphus isaea | 0 |  |
| H-I47-2J | P. sativum | Scaptomyza flava | Diglyphus isaea | Diglyphus isaea | Mini, ZBJ | Chromatomyia horticola |
| H-I47-2K | P. sativum | Scaptomyza flava | Diglyphus isaea | Diglyphus isaea | 0 |  |
| H-I47-2Oa | P. sativum | Scaptomyza flava | Diglyphus isaea | Diglyphus isaea | 0 |  |
| H-I47-2Ob | P. sativum | Scaptomyza flava | Diglyphus isaea | Diglyphus isaea | 0 |  |
| H-I47-2T | P. sativum | Scaptomyza flava | Diglyphus isaea | Diglyphus isaea | 0 |  |
| H-I47-2U | P. sativum | Scaptomyza flava | Diglyphus isaea | Diglyphus isaea | Mini | Chromatomyia horticola |
| H-I27-1J | P. sativum | Chromatomyia horticola | Chrysocharis pubicornis | Eulophidea sp. | Mini, ZBJ | Chromatomyia horticola |
| H-I47-2ADa | P. sativum | Scaptomyza flava | Chrysocharis pallipes | Eulophidea sp. | 0 |  |
| H-I47-2ADb | P. sativum | Scaptomyza flava | Chrysocharis pallipes | Eulophidea sp. | 0 |  |
| H-I47-2AFa | P. sativum | Scaptomyza flava | Chrysocharis pallipes | Eulophidea sp. | Mini, ZBJ | Chromatomyia syngenesiae |
| H-I47-2AFb | P. sativum | Scaptomyza flava | Chrysocharis pallipes | Eulophidea sp. | 0 |  |
| H-I47-2AHa | P. sativum | Scaptomyza flava | Chrysocharis pallipes | Eulophidea sp. | 0 |  |
| H-I47-2AHb | P. sativum | Scaptomyza flava | Chrysocharis pubicornis | Eulophidea sp. | Mini, ZBJ | Chromatomyia horticola |
| H-I47-2AIa | P. sativum | Scaptomyza flava | Chrysocharis pallipes | Eulophidea sp. | 0 |  |
| H-I47-2AIb | P. sativum | Scaptomyza flava | Chrysocharis pallipes | Eulophidea sp. | Mini, ZBJ | Chromatomyia horticola |
| H-I47-2AJ | P. sativum | Scaptomyza flava | Chrysocharis pallipes | Eulophidea sp. | 0 |  |
| H-I47-2AK | P. sativum | Scaptomyza flava | Chrysocharis pallipes | Eulophidea sp. | 0 |  |
| H-I47-2ANa | P. sativum | Scaptomyza flava | Chrysocharis pallipes | Eulophidea sp. | Mini, ZBJ | Chromatomyia horticola |
| H-I47-2ANb | P. sativum | Scaptomyza flava | Chrysocharis pallipes | Eulophidea sp. | 0 |  |
| H-I47-2AO | P. sativum | Scaptomyza flava | Chrysocharis pallipes | Eulophidea sp. | 0 |  |
| H-I47-2ASa | P. sativum | Scaptomyza flava | Chrysocharis pallipes | Eulophidea sp. | 0 |  |
| H-I47-2ASb | P. sativum | Scaptomyza flava | Chrysocharis pallipes | Eulophidea sp. | 0 |  |
| H-I47-2ASc | P. sativum | Scaptomyza flava | Chrysocharis pallipes | Eulophidea sp. | Mini, ZBJ | Chromatomyia syngenesiae |
| H-I47-2I | P. sativum | Scaptomyza flava | Chrysocharis pallipes | Eulophidea sp. | 0 |  |
| H-I47-2L | P. sativum | Scaptomyza flava | Chrysocharis pubicornis | Eulophidea sp. | Mini, ZBJ | Chromatomyia horticola |
| H-I47-2Qa | P. sativum | Scaptomyza flava | Chrysocharis pallipes | Eulophidea sp. | 0 |  |
| H-I47-2Qb | P. sativum | Scaptomyza flava | Chrysocharis pallipes | Eulophidea sp. | 0 |  |
| H-I47-2WB | P. sativum | Scaptomyza flava | Chrysocharis pallipes | Eulophidea sp. | 0 |  |
| H-I47-2X | P. sativum | Scaptomyza flava | Chrysocharis pallipes | Eulophidea sp. | 0 |  |
| H-I47-2Z | P. sativum | Scaptomyza flava | Chrysocharis pallipes | Eulophidea sp. | Mini, ZBJ | Chromatomyia horticola |
| H-I47-2AG | P. sativum | Scaptomyza flava | Halticoptera sp. | Hymenoptera sp. | 0 |  |

**Table S2. Comparison of three methods of molecular identification: BOLD similarity, Genbank megaBLAST and Maximum Likelihood Tree.**

| Voucher number | BOLD similarity |  | Genbank | Query coverage | Identity | ML Tree |
| --- | --- | --- | --- | --- | --- | --- |
| I33-2C | Chromatomyia nigra | 99.74 | Chromatomyia horticola | 98 | 95 | Chromatomyia nigra |
| I47-2AQ | Scaptomyza flava | 100 | Scaptomyza flava | 100 | 98 | Scaptomyza flava |
| I90-1 | Pegomyia solennis | 99.81 | Emmesomyia grisea | 99 | 93 | Pegomyia solennis |
| I27-1G | Chromatomyia horticola | 100 | Chromatomyia horticola | 98 | 99 | Chromatomyia horticola |
| I47-2AE | Scaptomyza flava | 100 | Scaptomyza flava | 100 | 98 | Scaptomyza flava |
| I33-2A | Chromatomyia syngenesiae | 99.64 | Chromatomyia syngenesiae | 94 | 99 | Chromatomyia syngenesiae |
| I27-1D | Scaptomyza flava | 100 | Scaptomyza flava | 100 | 98 | Scaptomyza flava |
| I23-4C | Chromatomyia nigra | 100 | Chromatomyia horticola | 100 | 100 | Chromatomyia horticola |
| I88-1 | Phytomyza spondylii | 99.83 | Phytomyza spondylii | 100 | 98 | Phytomyza spondylii |
| I90-1a | Pegomyia solennis | 99.5 | Calliphora quadrimaculata | 100 | 92 | Pegomyia solennis |
| I47-2B | Scaptomyza flava | 100 | Scaptomyza flava | 99 | 98 | Scaptomyza flava |
| I47-2AEa | Scaptomyza flava | 99.83 | Scaptomyza flava | 100 | 98 | Scaptomyza flava |
| I47-2D | Chromatomyia horticola | 100 | Chromatomyia horticola | 100 | 99 | Chromatomyia horticola |
| I33-2B | Chromatomyia syngenesiae | 99.48 | Chromatomyia syngenesiae | 94 | 99 | Chromatomyia syngenesiae |
| I44-22AEb | Chromatomyia syngenesiae | 99.46 | Chromatomyia syngenesiae | 91 | 99 | Chromatomyia syngenesiae |
| I90-1B | Pegomyia solennis | 99.78 | Emmesomyia grisea | 99 | 93 | Pegomyia solennis |
| I47-22Q | Scaptomyza flava | 100 | Scaptomyza flava | 98 | 98 | Scaptomyza flava |
| I47-2AL | Chromatomyia horticola | 100 | Chromatomyia horticola | 99 | 99 | Chromatomyia horticola |
| I47-2S | Chromatomyia horticola | 100 | Chromatomyia horticola | 97 | 99 | Chromatomyia horticola |
| I27-1I | Scaptomyza flava | 100 | Scaptomyza flava | 98 | 98 | Scaptomyza flava |
| I33-2Ca | Chromatomyia syngenesiae | 99.48 | Chromatomyia syngenesiae | 94 | 99 | Chromatomyia syngenesiae |
| I47-2AEc | Chromatomyia syngenesiae | 99.46 | Chromatomyia syngenesiae | 91 | 99 | Chromatomyia syngenesiae |
| I47-2AA | Chromatomyia horticola | 100 | Chromatomyia horticola | 100 | 100 | Chromatomyia horticola |
| I27-1Ia | Scaptomyza flava | 100 | Scaptomyza flava | 100 | 98 | Scaptomyza flava |
| I33-2Cb | Chromatomyia syngenesiae | 99.48 | Chromatomyia syngenesiae | 94 | 99 | Chromatomyia syngenesiae |
| I47-22AP | Chromatomyia syngenesiae | 99.48 | Chromatomyia syngenesiae | 94 | 99 | Chromatomyia syngenesiae |
| I27-1H | Chromatomyia horticola | 100 | Chromatomyia horticola | 100 | 99 | Chromatomyia horticola |
| I47-2M | Chromatomyia horticola | 100 | Chromatomyia horticola | 99 | 99 | Chromatomyia horticola |
| I27-1F | Chromatomyia horticola | 100 | Chromatomyia horticola | 99 | 99 | Chromatomyia horticola |
| I47-2ALa | Scaptomyza flava | 100 | Scaptomyza flava | 99 | 98 | Scaptomyza flava |
| I47-2ALb | Scaptomyza flava | 100 | Scaptomyza flava | 99 | 98 | Scaptomyza flava |
| I50-1G | Phytomyza spondylii | 100 | Phytomyza spondylii | 100 | 100 | Phytomyza spondylii |
| I47-2AAa | Chromatomyia horticola | 100 | Chromatomyia horticola | 99 | 99 | Chromatomyia horticola |
| I47-2APa | Chromatomyia syngenesiae | 99.48 | Chromatomyia syngenesiae | 94 | 99 | Chromatomyia syngenesiae |
| I47-2R | Chromatomyia horticola | 100 | Chromatomyia horticola | 99 | 100 | Chromatomyia horticola |
| I50-1Ga | Phytomyza spondylii | 100 | Phytomyza spondylii | 99 | 98 | Phytomyza spondylii |
| I47-2AB | Scaptomyza flava | 100 | Scaptomyza flava | 99 | 98 | Scaptomyza flava |
| I47-2AR | Scaptomyza flava | 100 | Scaptomyza flava | 100 | 98 | Scaptomyza flava |
| I50-1A | Phytomyza spondylii | 100 | Phytomyza spondylii | 100 | 100 | Phytomyza spondylii |
| I83-2 | Pegomyia solennis | 99.45 | Emmesomyia grisea | 94 | 93 | Pegomyia solennis |
| I47-2G | Scaptomyza flava | 100 | Scaptomyza flava | 100 | 98 | Scaptomyza flava |
| I47-2ABa | Scaptomyza flava | 100 | Scaptomyza flava | 100 | 98 | Scaptomyza flava |
| I47-2O | Chromatomyia horticola | 100 | Chromatomyia horticola | 100 | 100 | Chromatomyia horticola |
| I9-2B | Pegomyia bicolor | 100 | Anthomyiidae sp | 100 | 100 | Pegomyia bicolor |
| I23-4A | Scaptomyza flava | 100 | Scaptomyza flava | 100 | 98 | Scaptomyza flava |
| I47-2AEd | Scaptomyza flava | 100 | Scaptomyza flava | 100 | 98 | Scaptomyza flava |
| I23-4Aa | Chromatomyia horticola | 100 | Chromatomyia horticola | 99 | 99 | Chromatomyia horticola |
| I47-2AM | Scaptomyza flava | 100 | Scaptomyza flava | 99 | 99 | Scaptomyza flava |
| I33-2Cc | Chromatomyia syngenesiae | 99.48 | Chromatomyia syngenesiae | 94 | 99 | Chromatomyia syngenesiae |
| I47-2T | Scaptomyza flava | 100 | Scaptomyza flava | 100 | 98 | Scaptomyza flava |
| I47-2ARa | Scaptomyza flava | 100 | Scaptomyza flava | 100 | 98 | Scaptomyza flava |
| I23-4E | Scaptomyza flava | 100 | Scaptomyza flava | 100 | 98 | Scaptomyza flava |
| I23-4Ab | Scaptomyza flava | 100 | Scaptomyza flava | 100 | 98 | Scaptomyza flava |
| I47-2AEe | Scaptomyza flava | 100 | Scaptomyza flava | 100 | 98 | Scaptomyza flava |
| I27-1Da | Scaptomyza flava | 100 | Scaptomyza flava | 100 | 98 | Scaptomyza flava |
| I27-1E | Scaptomyza flava | 100 | Scaptomyza flava | 100 | 98 | Scaptomyza flava |
| I47-2Ga | Chromatomyia horticola | 100 | Chromatomyia horticola | 100 | 99 | Chromatomyia horticola |
| I23-4Ca | Scaptomyza flava | 100 | Scaptomyza flava | 100 | 98 | Scaptomyza flava |
| I23-4Cb | Chromatomyia horticola | 100 | Chromatomyia horticola | 100 | 100 | Chromatomyia horticola |
| I23-4B | Scaptomyza flava | 100 | Scaptomyza flava | 100 | 98 | Scaptomyza flava |
| I50-1E | Phytomyza spondylii | 100 | Phytomyza spondylii | 100 | 100 | Phytomyza spondylii |
| I23-4C | Scaptomyza flava | 100 | Scaptomyza flava | 100 | 98 | Scaptomyza flava |
| I87-3 | Pegomyia solennis | 99.67 | Scathophaga stercoraria | 99 | 92 | Pegomyia solennis |
| I87-3a | Pegomyia solennis | 99.67 | Scathophaga stercoraria | 99 | 92 | Pegomyia solennis |
| I86-1 | Cameraria ohridella | 100 | Cameraria ohridella | 100 | 100 | Cameraria ohridella |
| I60-1 | Agromyza frontella | 100 | Agromyza frontella | 100 | 99 | Agromyza frontella |
| I14-1 | Amauromyza flavifrons | 100 | Amauromyza flavifrons | 100 | 100 | Amauromyza flavifrons |
| I3-2C | Chromatomyia nigra | 100 | Chromatomyia horticola | 98 | 95 | Chromatomyia nigra |
| I7-1A | Chromatomyia nigra | 100 | Chromatomyia horticola | 98 | 95 | Chromatomyia nigra |
| I29-3 | Chromatomyia nigra | 100 | Chromatomyia horticola | 98 | 95 | Chromatomyia nigra |
| I10-2C | Chromatomyia horticola | 100 | Chromatomyia horticola | 100 | 99 | Chromatomyia horticola |
| I10-2E | Chromatomyia horticola | 100 | Chromatomyia horticola | 100 | 99 | Chromatomyia horticola |
| I10-2K | Chromatomyia horticola | 100 | Chromatomyia horticola | 100 | 99 | Chromatomyia horticola |
| I1-2B | Chromatomyia horticola | 100 | Chromatomyia horticola | 100 | 99 | Chromatomyia horticola |
| I3-2E | Chromatomyia horticola | 100 | Chromatomyia horticola | 100 | 99 | Chromatomyia horticola |
| I3-2G | Chromatomyia horticola | 100 | Chromatomyia horticola | 100 | 99 | Chromatomyia horticola |
| I9-1D | Chromatomyia horticola | 100 | Chromatomyia horticola | 100 | 99 | Chromatomyia horticola |
| I9-1E | Chromatomyia horticola | 100 | Chromatomyia horticola | 100 | 99 | Chromatomyia horticola |
| I9-1F | Chromatomyia horticola | 100 | Chromatomyia horticola | 100 | 99 | Chromatomyia horticola |
| I19-1A | Chromatomyia horticola | 100 | Chromatomyia horticola | 100 | 99 | Chromatomyia horticola |
| I19-1C | Chromatomyia horticola | 100 | Chromatomyia horticola | 100 | 99 | Chromatomyia horticola |
| I19-1F | Chromatomyia horticola | 100 | Chromatomyia horticola | 100 | 99 | Chromatomyia horticola |
| I19-1G | Chromatomyia horticola | 100 | Chromatomyia horticola | 100 | 99 | Chromatomyia horticola |
| I20-1C | Chromatomyia horticola | 100 | Chromatomyia horticola | 100 | 99 | Chromatomyia horticola |
| I21-3B | Chromatomyia horticola | 100 | Chromatomyia horticola | 100 | 99 | Chromatomyia horticola |
| I21-3E | Chromatomyia horticola | 100 | Chromatomyia horticola | 100 | 99 | Chromatomyia horticola |
| I21-3C | Chromatomyia horticola | 100 | Chromatomyia horticola | 100 | 99 | Chromatomyia horticola |
| I21-3D | Chromatomyia horticola | 100 | Chromatomyia horticola | 100 | 99 | Chromatomyia horticola |
| I21-3F | Chromatomyia horticola | 100 | Chromatomyia horticola | 100 | 99 | Chromatomyia horticola |
| I9-1D | Chrysoesthia drurella | 100 | Chrysoesthia drurella | 100 | 100 | Chrysoesthia drurella |
| I10-2B | Chrysoesthia drurella | 100 | Chrysoesthia drurella | 100 | 100 | Chrysoesthia drurella |
| I6-1C | Stigmella samiatella | 100 | Stigmella samiatella | 100 | 100 | Stigmella samiatella |
| I81-1 | Ectoedemia albifasciella | 100 | Ectoedemia albifasciella | 100 | 100 | Ectoedemia albifasciella |
| I10-1A | Phytomyza cirsii | 100 | Phytomyza cirsii | 100 | 100 | Phytomyza cirsii |
| I85-1 | Cameraria ohridella | 100 | Cameraria ohridella | 100 | 100 | Cameraria ohridella |
| I87-2 | Coleophora gryphipennella | 100 | Coleophora gryphipennella | 100 | 99 | Coleophora gryphipennella |
| I87-1 | Coleophora gryphipennella | 100 | Coleophora gryphipennella | 100 | 99 | Coleophora gryphipennella |
| I90-2 | Stigmella splendidissimella | 100 | Stigmella splendidissimella | 99 | 100 | Stigmella splendidissimella |
| I85-4 | Stigmella splendidissimella | 100 | Stigmella splendidissimella | 99 | 100 | Stigmella splendidissimella |
| I47-2AEf | Braconidae | 93.3 | Dacnusa sibirica | 98 | 92 | Braconidae sp |
| I27-1Ib | Braconidae | 93.4 | Chorebus sp | 100 | 92 | Braconidae sp |
| I23-3EC | Braconidae | 95.4 | Alysiinae sp | 98 | 92 | Braconidae sp |
| I23-4E | Braconidae | 93.4 | Chorebus sp | 100 | 92 | Braconidae sp |
| I23-4EB | Braconidae | 93.4 | Chorebus sp | 100 | 92 | Braconidae sp |
| I23-4D | Braconidae | 93.4 | Chorebus sp | 100 | 92 | Braconidae sp |
| I47-2W | Braconidae | 93.4 | Chorebus sp | 100 | 92 | Braconidae sp |
| I47-2AM | Braconidae | 93.4 | Chorebus sp | 100 | 92 | Braconidae sp |
| I23-4B | Braconidae | 93.4 | Chorebus sp | 100 | 92 | Braconidae sp |
| I27-1C | Braconidae | 93.4 | Chorebus sp | 100 | 92 | Braconidae sp |
| I27-1G | Braconidae | 93.4 | Chorebus sp | 100 | 92 | Braconidae sp |
| I47-2AL | Dacnusa sp | 100 | Dacnusa sibirica | 100 | 94 | Dacnusa sp |
| I47-2K | Dygliphus isaea | 98.66 | Dygliphus isaea | 100 | 99 | Dygliphus isaea |
| I47-2J | Dygliphus isaea | 99.5 | Dygliphus isaea | 100 | 100 | Dygliphus isaea |
| I47-2G | Dygliphus isaea | 98.35 | Dygliphus isaea | 100 | 98 | Dygliphus isaea |
| I47-2T | Dygliphus isaea | 98.5 | Dygliphus isaea | 100 | 98 | Dygliphus isaea |
| I21-3Aa | Dygliphus isaea | 98 | Dygliphus isaea | 100 | 98 | Dygliphus isaea |
| I47-2Oa | Dygliphus isaea | 98.45 | Dygliphus isaea | 100 | 99 | Dygliphus isaea |
| I21-3E | Dygliphus isaea | 98.34 | Dygliphus isaea | 100 | 99 | Dygliphus isaea |
| I47-2F | Dygliphus isaea | 98.63 | Dygliphus isaea | 100 | 99 | Dygliphus isaea |
| I21-3Ab | Dygliphus isaea | 99.14 | Dygliphus isaea | 100 | 99 | Dygliphus isaea |
| I47-2U | Dygliphus isaea | 98.8 | Dygliphus isaea | 100 | 99 | Dygliphus isaea |
| I47-2Ob | Dygliphus isaea | 98.8 | Dygliphus isaea | 100 | 99 | Dygliphus isaea |
| I47-2WB | Eulophidae sp | 99.5 | Eulophidae sp | 97 | 89 | Eulophidae sp |
| I47-2ANa | Eulophidae sp | 99.5 | Eulophidae sp | 97 | 89 | Eulophidae sp |
| I47-2AIa | Eulophidae sp | 99.5 | Eulophidae sp | 97 | 89 | Eulophidae sp |
| I47-2X | Eulophidae sp | 99.5 | Eulophidae sp | 97 | 89 | Eulophidae sp |
| I47-2I | Eulophidae sp | 99.5 | Eulophidae sp | 97 | 89 | Eulophidae sp |
| I47-2AHa | Eulophidae sp | 99.5 | Eulophidae sp | 97 | 89 | Eulophidae sp |
| I47-2AJ | Eulophidae sp | 99.5 | Eulophidae sp | 97 | 89 | Eulophidae sp |
| I47-2AO | Eulophidae sp | 99.5 | Eulophidae sp | 97 | 89 | Eulophidae sp |
| I47-2Z | Eulophidae sp | 99.5 | Eulophidae sp | 97 | 89 | Eulophidae sp |
| I47-2ASa | Eulophidae sp | 99.5 | Eulophidae sp | 97 | 89 | Eulophidae sp |
| I47-2ADa | Eulophidae sp | 99.5 | Eulophidae sp | 97 | 89 | Eulophidae sp |
| I47-22ASb | Eulophidae sp | 99.5 | Eulophidae sp | 97 | 89 | Eulophidae sp |
| I47-2Qa | Eulophidae sp | 99.5 | Eulophidae sp | 97 | 89 | Eulophidae sp |
| I47-2ADb | Eulophidae sp | 99.5 | Eulophidae sp | 97 | 89 | Eulophidae sp |
| I47-2ASc | Eulophidae sp | 99.5 | Eulophidae sp | 97 | 89 | Eulophidae sp |
| I47-2Qb | Eulophidae sp | 99.5 | Eulophidae sp | 97 | 89 | Eulophidae sp |
| I47-2AFa | Eulophidae sp | 99.5 | Eulophidae sp | 97 | 89 | Eulophidae sp |
| I47-2AFb | Eulophidae sp | 99.5 | Eulophidae sp | 97 | 89 | Eulophidae sp |
| I47-2ANb | Eulophidae sp | 99.5 | Eulophidae sp | 97 | 89 | Eulophidae sp |
| I47-2AG | Hymenoptera sp | 90.99 | Hymenoptera sp | 97 | 86 | Hymenoptera sp |
| I47-2AIb | Eulophidae sp | 99.5 | Eulophidae sp | 97 | 89 | Eulophidae sp |
| I47-2AHb | Hymenoptera sp | 97.8 | Achrysocharoides sp | 98 | 89 | Hymenoptera sp |
| I47-2L | Hymenoptera sp | 97.8 | Achrysocharoides sp | 98 | 89 | Hymenoptera sp |
| I27-1J | Hymenoptera sp | 97.8 | Achrysocharoides sp | 98 | 89 | Hymenoptera sp |
| I47-2AK | Eulophidae sp | 99.5 | Eulophidae sp | 97 | 89 | Eulophidae sp |
