# Supplementary material for: Determining Plant – Leaf Miner – Parasitoid Interactions: A DNA Barcoding Approach
Source: PLoS One. 2015 Feb 24;10(2):e0117872. doi: 10.1371/journal.pone.0117872 (PMC4339730; doi:10.1371/journal.pone.0117872)
Supplement: S2 Table — (DOCX) [file pone.0117872.s002.docx]

**Supporting Information**

**Table S2. Comparison of three methods of molecular identification: BOLD similarity, Genbank megaBLAST and Maximum Likelihood Tree.**

| Voucher number | BOLD similarity |  | Genbank | Query coverage | Identity | ML Tree |
| --- | --- | --- | --- | --- | --- | --- |
| I33-2C | Chromatomyia nigra | 99.74 | Chromatomyia horticola | 98 | 95 | Chromatomyia nigra |
| I47-2AQ | Scaptomyza flava | 100 | Scaptomyza flava | 100 | 98 | Scaptomyza flava |
| I90-1 | Pegomyia solennis | 99.81 | Emmesomyia grisea | 99 | 93 | Pegomyia solennis |
| I27-1G | Chromatomyia horticola | 100 | Chromatomyia horticola | 98 | 99 | Chromatomyia horticola |
| I47-2AE | Scaptomyza flava | 100 | Scaptomyza flava | 100 | 98 | Scaptomyza flava |
| I33-2A | Chromatomyia syngenesiae | 99.64 | Chromatomyia syngenesiae | 94 | 99 | Chromatomyia syngenesiae |
| I27-1D | Scaptomyza flava | 100 | Scaptomyza flava | 100 | 98 | Scaptomyza flava |
| I23-4C | Chromatomyia nigra | 100 | Chromatomyia horticola | 100 | 100 | Chromatomyia horticola |
| I88-1 | Phytomyza spondylii | 99.83 | Phytomyza spondylii | 100 | 98 | Phytomyza spondylii |
| I90-1a | Pegomyia solennis | 99.5 | Calliphora quadrimaculata | 100 | 92 | Pegomyia solennis |
| I47-2B | Scaptomyza flava | 100 | Scaptomyza flava | 99 | 98 | Scaptomyza flava |
| I47-2AEa | Scaptomyza flava | 99.83 | Scaptomyza flava | 100 | 98 | Scaptomyza flava |
| I47-2D | Chromatomyia horticola | 100 | Chromatomyia horticola | 100 | 99 | Chromatomyia horticola |
| I33-2B | Chromatomyia syngenesiae | 99.48 | Chromatomyia syngenesiae | 94 | 99 | Chromatomyia syngenesiae |
| I44-22AEb | Chromatomyia syngenesiae | 99.46 | Chromatomyia syngenesiae | 91 | 99 | Chromatomyia syngenesiae |
| I90-1B | Pegomyia solennis | 99.78 | Emmesomyia grisea | 99 | 93 | Pegomyia solennis |
| I47-22Q | Scaptomyza flava | 100 | Scaptomyza flava | 98 | 98 | Scaptomyza flava |
| I47-2AL | Chromatomyia horticola | 100 | Chromatomyia horticola | 99 | 99 | Chromatomyia horticola |
| I47-2S | Chromatomyia horticola | 100 | Chromatomyia horticola | 97 | 99 | Chromatomyia horticola |
| I27-1I | Scaptomyza flava | 100 | Scaptomyza flava | 98 | 98 | Scaptomyza flava |
| I33-2Ca | Chromatomyia syngenesiae | 99.48 | Chromatomyia syngenesiae | 94 | 99 | Chromatomyia syngenesiae |
| I47-2AEc | Chromatomyia syngenesiae | 99.46 | Chromatomyia syngenesiae | 91 | 99 | Chromatomyia syngenesiae |
| I47-2AA | Chromatomyia horticola | 100 | Chromatomyia horticola | 100 | 100 | Chromatomyia horticola |
| I27-1Ia | Scaptomyza flava | 100 | Scaptomyza flava | 100 | 98 | Scaptomyza flava |
| I33-2Cb | Chromatomyia syngenesiae | 99.48 | Chromatomyia syngenesiae | 94 | 99 | Chromatomyia syngenesiae |
| I47-22AP | Chromatomyia syngenesiae | 99.48 | Chromatomyia syngenesiae | 94 | 99 | Chromatomyia syngenesiae |
| I27-1H | Chromatomyia horticola | 100 | Chromatomyia horticola | 100 | 99 | Chromatomyia horticola |
| I47-2M | Chromatomyia horticola | 100 | Chromatomyia horticola | 99 | 99 | Chromatomyia horticola |
| I27-1F | Chromatomyia horticola | 100 | Chromatomyia horticola | 99 | 99 | Chromatomyia horticola |
| I47-2ALa | Scaptomyza flava | 100 | Scaptomyza flava | 99 | 98 | Scaptomyza flava |
| I47-2ALb | Scaptomyza flava | 100 | Scaptomyza flava | 99 | 98 | Scaptomyza flava |
| I50-1G | Phytomyza spondylii | 100 | Phytomyza spondylii | 100 | 100 | Phytomyza spondylii |
| I47-2AAa | Chromatomyia horticola | 100 | Chromatomyia horticola | 99 | 99 | Chromatomyia horticola |
| I47-2APa | Chromatomyia syngenesiae | 99.48 | Chromatomyia syngenesiae | 94 | 99 | Chromatomyia syngenesiae |
| I47-2R | Chromatomyia horticola | 100 | Chromatomyia horticola | 99 | 100 | Chromatomyia horticola |
| I50-1Ga | Phytomyza spondylii | 100 | Phytomyza spondylii | 99 | 98 | Phytomyza spondylii |
| I47-2AB | Scaptomyza flava | 100 | Scaptomyza flava | 99 | 98 | Scaptomyza flava |
| I47-2AR | Scaptomyza flava | 100 | Scaptomyza flava | 100 | 98 | Scaptomyza flava |
| I50-1A | Phytomyza spondylii | 100 | Phytomyza spondylii | 100 | 100 | Phytomyza spondylii |
| I83-2 | Pegomyia solennis | 99.45 | Emmesomyia grisea | 94 | 93 | Pegomyia solennis |
| I47-2G | Scaptomyza flava | 100 | Scaptomyza flava | 100 | 98 | Scaptomyza flava |
| I47-2ABa | Scaptomyza flava | 100 | Scaptomyza flava | 100 | 98 | Scaptomyza flava |
| I47-2O | Chromatomyia horticola | 100 | Chromatomyia horticola | 100 | 100 | Chromatomyia horticola |
| I9-2B | Pegomyia bicolor | 100 | Anthomyiidae sp | 100 | 100 | Pegomyia bicolor |
| I23-4A | Scaptomyza flava | 100 | Scaptomyza flava | 100 | 98 | Scaptomyza flava |
| I47-2AEd | Scaptomyza flava | 100 | Scaptomyza flava | 100 | 98 | Scaptomyza flava |
| I23-4Aa | Chromatomyia horticola | 100 | Chromatomyia horticola | 99 | 99 | Chromatomyia horticola |
| I47-2AM | Scaptomyza flava | 100 | Scaptomyza flava | 99 | 99 | Scaptomyza flava |
| I33-2Cc | Chromatomyia syngenesiae | 99.48 | Chromatomyia syngenesiae | 94 | 99 | Chromatomyia syngenesiae |
| I47-2T | Scaptomyza flava | 100 | Scaptomyza flava | 100 | 98 | Scaptomyza flava |
| I47-2ARa | Scaptomyza flava | 100 | Scaptomyza flava | 100 | 98 | Scaptomyza flava |
| I23-4E | Scaptomyza flava | 100 | Scaptomyza flava | 100 | 98 | Scaptomyza flava |
| I23-4Ab | Scaptomyza flava | 100 | Scaptomyza flava | 100 | 98 | Scaptomyza flava |
| I47-2AEe | Scaptomyza flava | 100 | Scaptomyza flava | 100 | 98 | Scaptomyza flava |
| I27-1Da | Scaptomyza flava | 100 | Scaptomyza flava | 100 | 98 | Scaptomyza flava |
| I27-1E | Scaptomyza flava | 100 | Scaptomyza flava | 100 | 98 | Scaptomyza flava |
| I47-2Ga | Chromatomyia horticola | 100 | Chromatomyia horticola | 100 | 99 | Chromatomyia horticola |
| I23-4Ca | Scaptomyza flava | 100 | Scaptomyza flava | 100 | 98 | Scaptomyza flava |
| I23-4Cb | Chromatomyia horticola | 100 | Chromatomyia horticola | 100 | 100 | Chromatomyia horticola |
| I23-4B | Scaptomyza flava | 100 | Scaptomyza flava | 100 | 98 | Scaptomyza flava |
| I50-1E | Phytomyza spondylii | 100 | Phytomyza spondylii | 100 | 100 | Phytomyza spondylii |
| I23-4C | Scaptomyza flava | 100 | Scaptomyza flava | 100 | 98 | Scaptomyza flava |
| I87-3 | Pegomyia solennis | 99.67 | Scathophaga stercoraria | 99 | 92 | Pegomyia solennis |
| I87-3a | Pegomyia solennis | 99.67 | Scathophaga stercoraria | 99 | 92 | Pegomyia solennis |
| I86-1 | Cameraria ohridella | 100 | Cameraria ohridella | 100 | 100 | Cameraria ohridella |
| I60-1 | Agromyza frontella | 100 | Agromyza frontella | 100 | 99 | Agromyza frontella |
| I14-1 | Amauromyza flavifrons | 100 | Amauromyza flavifrons | 100 | 100 | Amauromyza flavifrons |
| I3-2C | Chromatomyia nigra | 100 | Chromatomyia horticola | 98 | 95 | Chromatomyia nigra |
| I7-1A | Chromatomyia nigra | 100 | Chromatomyia horticola | 98 | 95 | Chromatomyia nigra |
| I29-3 | Chromatomyia nigra | 100 | Chromatomyia horticola | 98 | 95 | Chromatomyia nigra |
| I10-2C | Chromatomyia horticola | 100 | Chromatomyia horticola | 100 | 99 | Chromatomyia horticola |
| I10-2E | Chromatomyia horticola | 100 | Chromatomyia horticola | 100 | 99 | Chromatomyia horticola |
| I10-2K | Chromatomyia horticola | 100 | Chromatomyia horticola | 100 | 99 | Chromatomyia horticola |
| I1-2B | Chromatomyia horticola | 100 | Chromatomyia horticola | 100 | 99 | Chromatomyia horticola |
| I3-2E | Chromatomyia horticola | 100 | Chromatomyia horticola | 100 | 99 | Chromatomyia horticola |
| I3-2G | Chromatomyia horticola | 100 | Chromatomyia horticola | 100 | 99 | Chromatomyia horticola |
| I9-1D | Chromatomyia horticola | 100 | Chromatomyia horticola | 100 | 99 | Chromatomyia horticola |
| I9-1E | Chromatomyia horticola | 100 | Chromatomyia horticola | 100 | 99 | Chromatomyia horticola |
| I9-1F | Chromatomyia horticola | 100 | Chromatomyia horticola | 100 | 99 | Chromatomyia horticola |
| I19-1A | Chromatomyia horticola | 100 | Chromatomyia horticola | 100 | 99 | Chromatomyia horticola |
| I19-1C | Chromatomyia horticola | 100 | Chromatomyia horticola | 100 | 99 | Chromatomyia horticola |
| I19-1F | Chromatomyia horticola | 100 | Chromatomyia horticola | 100 | 99 | Chromatomyia horticola |
| I19-1G | Chromatomyia horticola | 100 | Chromatomyia horticola | 100 | 99 | Chromatomyia horticola |
| I20-1C | Chromatomyia horticola | 100 | Chromatomyia horticola | 100 | 99 | Chromatomyia horticola |
| I21-3B | Chromatomyia horticola | 100 | Chromatomyia horticola | 100 | 99 | Chromatomyia horticola |
| I21-3E | Chromatomyia horticola | 100 | Chromatomyia horticola | 100 | 99 | Chromatomyia horticola |
| I21-3C | Chromatomyia horticola | 100 | Chromatomyia horticola | 100 | 99 | Chromatomyia horticola |
| I21-3D | Chromatomyia horticola | 100 | Chromatomyia horticola | 100 | 99 | Chromatomyia horticola |
| I21-3F | Chromatomyia horticola | 100 | Chromatomyia horticola | 100 | 99 | Chromatomyia horticola |
| I9-1D | Chrysoesthia drurella | 100 | Chrysoesthia drurella | 100 | 100 | Chrysoesthia drurella |
| I10-2B | Chrysoesthia drurella | 100 | Chrysoesthia drurella | 100 | 100 | Chrysoesthia drurella |
| I6-1C | Stigmella samiatella | 100 | Stigmella samiatella | 100 | 100 | Stigmella samiatella |
| I81-1 | Ectoedemia albifasciella | 100 | Ectoedemia albifasciella | 100 | 100 | Ectoedemia albifasciella |
| I10-1A | Phytomyza cirsii | 100 | Phytomyza cirsii | 100 | 100 | Phytomyza cirsii |
| I85-1 | Cameraria ohridella | 100 | Cameraria ohridella | 100 | 100 | Cameraria ohridella |
| I87-2 | Coleophora gryphipennella | 100 | Coleophora gryphipennella | 100 | 99 | Coleophora gryphipennella |
| I87-1 | Coleophora gryphipennella | 100 | Coleophora gryphipennella | 100 | 99 | Coleophora gryphipennella |
| I90-2 | Stigmella splendidissimella | 100 | Stigmella splendidissimella | 99 | 100 | Stigmella splendidissimella |
| I85-4 | Stigmella splendidissimella | 100 | Stigmella splendidissimella | 99 | 100 | Stigmella splendidissimella |
| I47-2AEf | Braconidae | 93.3 | Dacnusa sibirica | 98 | 92 | Braconidae sp |
| I27-1Ib | Braconidae | 93.4 | Chorebus sp | 100 | 92 | Braconidae sp |
| I23-3EC | Braconidae | 95.4 | Alysiinae sp | 98 | 92 | Braconidae sp |
| I23-4E | Braconidae | 93.4 | Chorebus sp | 100 | 92 | Braconidae sp |
| I23-4EB | Braconidae | 93.4 | Chorebus sp | 100 | 92 | Braconidae sp |
| I23-4D | Braconidae | 93.4 | Chorebus sp | 100 | 92 | Braconidae sp |
| I47-2W | Braconidae | 93.4 | Chorebus sp | 100 | 92 | Braconidae sp |
| I47-2AM | Braconidae | 93.4 | Chorebus sp | 100 | 92 | Braconidae sp |
| I23-4B | Braconidae | 93.4 | Chorebus sp | 100 | 92 | Braconidae sp |
| I27-1C | Braconidae | 93.4 | Chorebus sp | 100 | 92 | Braconidae sp |
| I27-1G | Braconidae | 93.4 | Chorebus sp | 100 | 92 | Braconidae sp |
| I47-2AL | Dacnusa sp | 100 | Dacnusa sibirica | 100 | 94 | Dacnusa sp |
| I47-2K | Dygliphus isaea | 98.66 | Dygliphus isaea | 100 | 99 | Dygliphus isaea |
| I47-2J | Dygliphus isaea | 99.5 | Dygliphus isaea | 100 | 100 | Dygliphus isaea |
| I47-2G | Dygliphus isaea | 98.35 | Dygliphus isaea | 100 | 98 | Dygliphus isaea |
| I47-2T | Dygliphus isaea | 98.5 | Dygliphus isaea | 100 | 98 | Dygliphus isaea |
| I21-3Aa | Dygliphus isaea | 98 | Dygliphus isaea | 100 | 98 | Dygliphus isaea |
| I47-2Oa | Dygliphus isaea | 98.45 | Dygliphus isaea | 100 | 99 | Dygliphus isaea |
| I21-3E | Dygliphus isaea | 98.34 | Dygliphus isaea | 100 | 99 | Dygliphus isaea |
| I47-2F | Dygliphus isaea | 98.63 | Dygliphus isaea | 100 | 99 | Dygliphus isaea |
| I21-3Ab | Dygliphus isaea | 99.14 | Dygliphus isaea | 100 | 99 | Dygliphus isaea |
| I47-2U | Dygliphus isaea | 98.8 | Dygliphus isaea | 100 | 99 | Dygliphus isaea |
| I47-2Ob | Dygliphus isaea | 98.8 | Dygliphus isaea | 100 | 99 | Dygliphus isaea |
| I47-2WB | Eulophidae sp | 99.5 | Eulophidae sp | 97 | 89 | Eulophidae sp |
| I47-2ANa | Eulophidae sp | 99.5 | Eulophidae sp | 97 | 89 | Eulophidae sp |
| I47-2AIa | Eulophidae sp | 99.5 | Eulophidae sp | 97 | 89 | Eulophidae sp |
| I47-2X | Eulophidae sp | 99.5 | Eulophidae sp | 97 | 89 | Eulophidae sp |
| I47-2I | Eulophidae sp | 99.5 | Eulophidae sp | 97 | 89 | Eulophidae sp |
| I47-2AHa | Eulophidae sp | 99.5 | Eulophidae sp | 97 | 89 | Eulophidae sp |
| I47-2AJ | Eulophidae sp | 99.5 | Eulophidae sp | 97 | 89 | Eulophidae sp |
| I47-2AO | Eulophidae sp | 99.5 | Eulophidae sp | 97 | 89 | Eulophidae sp |
| I47-2Z | Eulophidae sp | 99.5 | Eulophidae sp | 97 | 89 | Eulophidae sp |
| I47-2ASa | Eulophidae sp | 99.5 | Eulophidae sp | 97 | 89 | Eulophidae sp |
| I47-2ADa | Eulophidae sp | 99.5 | Eulophidae sp | 97 | 89 | Eulophidae sp |
| I47-22ASb | Eulophidae sp | 99.5 | Eulophidae sp | 97 | 89 | Eulophidae sp |
| I47-2Qa | Eulophidae sp | 99.5 | Eulophidae sp | 97 | 89 | Eulophidae sp |
| I47-2ADb | Eulophidae sp | 99.5 | Eulophidae sp | 97 | 89 | Eulophidae sp |
| I47-2ASc | Eulophidae sp | 99.5 | Eulophidae sp | 97 | 89 | Eulophidae sp |
| I47-2Qb | Eulophidae sp | 99.5 | Eulophidae sp | 97 | 89 | Eulophidae sp |
| I47-2AFa | Eulophidae sp | 99.5 | Eulophidae sp | 97 | 89 | Eulophidae sp |
| I47-2AFb | Eulophidae sp | 99.5 | Eulophidae sp | 97 | 89 | Eulophidae sp |
| I47-2ANb | Eulophidae sp | 99.5 | Eulophidae sp | 97 | 89 | Eulophidae sp |
| I47-2AG | Hymenoptera sp | 90.99 | Hymenoptera sp | 97 | 86 | Hymenoptera sp |
| I47-2AIb | Eulophidae sp | 99.5 | Eulophidae sp | 97 | 89 | Eulophidae sp |
| I47-2AHb | Hymenoptera sp | 97.8 | Achrysocharoides sp | 98 | 89 | Hymenoptera sp |
| I47-2L | Hymenoptera sp | 97.8 | Achrysocharoides sp | 98 | 89 | Hymenoptera sp |
| I27-1J | Hymenoptera sp | 97.8 | Achrysocharoides sp | 98 | 89 | Hymenoptera sp |
| I47-2AK | Eulophidae sp | 99.5 | Eulophidae sp | 97 | 89 | Eulophidae sp |
